# Computational Strategy for Analyzing Effective Properties of Random Composites—Part II: Elasticity

**DOI:** 10.3390/ma18215041

**Published:** 2025-11-05

**Authors:** Roman Czapla, Piotr Drygaś, Simon Gluzman, Tomasz Ligocki, Vladimir Mityushev

**Affiliations:** 1 Institute of Security and Computer Science, University of National Education Commission, Podchorążych 2, 30-084 Krakow, Poland; 2 Faculty of Computer Science and Telecommunications, Cracow University of Technology, Warszawska St., 24, 31-155 Krakow, Poland; piotr.drygas@pk.edu.pl (P.D.); wladimir.mitiuszew@pk.edu.pl (V.M.); 3 Materialica+ Research Group, Bathurst St. 3000, Apt. 606, Toronto, ON M6B 3B4, Canada; 4 Doctoral School, Faculty of Computer Science and Telecommunications, Cracow University of Technology, Warszawska St., 24, 31-155 Krakow, Poland; tomasz.ligocki@doktorant.pk.edu.pl

**Keywords:** Hill’s concept, homogenization, fibrous composite, computer simulation, RVE, resummation, critical index

## Abstract

We suggest a novel strategy in the theory of elastic plane composites. The macroscopic properties are quantified, and an analytical–numerical algorithm to derive expressions for the effective constants is designed. The effective elastic constants of dispersed random composites are given by new analytical and approximate formulas where the dependence on the location of inclusions is explicitly shown in symbolic form. This essentially extends the results of previous numerical simulations for a fixed set of material constants and fixed locations of inclusions. This paper extends the analysis from Part I, which addressed dispersed random conducting composites, to the two-dimensional elastic composites. Hill’s concept of Representative Volume Element (RVE), traditionally used in elastic composites, is revised. It is rigorously demonstrated that the RVE must be a fundamental domain of the plane torus, for instance, a periodicity parallelogram, since other shapes of RVE may lead to incorrect values of the effective constants. The effective tensors of the elasticity theory are decomposed into geometrical and physical parts, represented by structural sums and material constants of the components. Novel computational methodology based on such decomposition is applied to a two-phase isotropic composite with non-overlapping circular inclusions embedded in an elastic matrix. For the first time, it is demonstrated explicitly how the effective tensors depend on the geometric probabilistic distributions of inclusions and the computational protocols involved. Analytical polynomial formulas for the effective shear modulus for the moderate concentration of inclusions are transformed using the resummation methods into practical expressions valid for all concentrations of inclusions. The critical index for the effective shear modulus is calculated from the polynomials derived for the modulus.

## 1. Introduction

In this paper, the study of random conducting dispersed composites [1], Part I, is extended to two-dimensional (2D) elastic composites. The approach is transferable since stationary conductivity (permittivity, diffusivity, etc.) and antiplane strain in fibrous composites follow the same governing equations. The methodology is deeply entrenched in mathematical homogenization theory. Its framework was established solidly in [1]. This leads to a novel strategy for computing effective elastic constants for dispersed random composite materials. The strategy is constructive due to the specific constraints imposed in theory and simulations.

In the 1980s, the mathematical principles of homogenization theory were firmly established. Various subjects on homogenization are covered in the books [2,3,4,5]; see also multiple references therein. However, physicists and engineers applied periodic boundary problems to calculate the effective properties of composites much earlier, beginning from Rayleigh’s seminal paper [6] concerning the conductivity (or antiplane deformation). Natanzon [7] and Filshtinsky [8,9] considered plane elastic composites. Historical notes and references can be found in [10].

Maxwell’s self-consistent approach [11] to conductivity problems initiated a separate line of research in the theory of composites. The region of its validity and asymptotic justification can be found in [12,13]. Hill’s self-consistent conception [14,15], closely related to Maxwell’s approach, was proposed in 1963 for elastic composites. It was further extended in many works, such as [16]. Hill [14] introduced the notion of the Representative Volume Element (RVE) based on the self-consistent conception of a composite. This conception was discussed and revised in [1,17] for antiplane problems. The homogenization method and the self-consistent method consider different representative cells and produce different results. The elasticity problem requires a separate investigation of the correct choice of representative cells; see the discussion in Section 4.

In 1963, Hill proposed the concept of RVE, summarized in the following description [14]. “*Representative volume.* This phrase will be used when referring to a sample that (a) is structurally entirely typical of the whole mixture as long as its average properties are concerned; and (b) contains a sufficient number of inclusions for the apparent overall moduli to be effectively independent of the surface values of traction and displacement, so long as these values are “macroscopically uniform”. That is, they fluctuate about a mean (sic) with a wavelength small compared with the dimensions of the sample, and the effects of such fluctuations become insignificant within a few wavelengths of the surface. The contribution of this surface layer to any average can be made negligible (sic) by studying the sample that is large enough”.

Hill’s definition is undoubtedly rather valuable. It is often accepted by engineers as a definition of RVE and can be considered as an intuitive physical postulate behind the mathematical RVE theory. Yet, we do not have here a rigorous mathematical definition based on abstract quantifiers. The existing definitions are better understood de facto as methods for calculating the effective constants for a sufficiently large number of inclusions. However, they are not sufficient for random composites when a probabilistic distribution must be introduced precisely and follow the homogenization principles.

Hill [15] proposed to use the linear displacement and uniform traction boundary value problem in the form of (20) and (21), for a domain *V*, to determine the effective properties represented by the heterogeneous element *V*. Therefore, Hill proposed the concept [14], the method [15], and the motivation to investigate dispersed elastic composites. Hill managed to do it well before the era of homogenization had begun. His approach was applied by using numerical simulations but was not rigorously justified.

We proceed now with the discussion of pure numerical methods and their effectiveness for random elastic-dispersed 2D composites. The finite element method (FEM) is the most popular numerical method [18,19]. Accordingly, some advanced numerical packages have been developed. The user only has to introduce the corresponding geometric and mechanical data characterizing the elastic problem and obtain the output in the form of local elastic fields and effective elastic tensors [20]. This approach is computationally efficient when applied to digitalized real pictures taken in the course of experiments and used in morphological models. The geometric data are transformed into numerical form. The material constants must also be given numerically.

However, there are essential restrictions for the practical use of these packages for random composites. In particular, any practical implementation of randomness should be built on a solid statistical foundation. Most importantly, there are infinitely many probabilistic distributions that determine the classes and the corresponding effective properties of composites. The independent and identically distributed (i.i.d.) disks satisfy the uniform distribution, which is used tacitly in engineering works. In addition, the implementation of the simplest uniform distribution of equal disks requires solving about 1000 problems to achieve high precision, each having N=100 inclusions per cell [21]. A comprehensive investigation for a two-phase composite requires computation for a wide set of constants μ, ν, and $\mu_{1}$, $\nu_{1}$. In many realistic composites, thorough investigations are required for the number of inclusions about $N\approx 10^{6}$ [22,23]. Modern computers cannot achieve this goal by straightforward application of standard FEM packages. This makes their application for a random composite doubtful because of huge computational costs, especially when the total amount of time is considered. A discussion of efficient computationally oriented models of various systems using asymptotic methods that do not involve FEM can be found in [24]. At the same time, FEM is highly effective for fixed geometry and given material constants as a “single-use”.

The method of integral equations [9,25,26] seems to be more efficient for the calculation of effective constants. It is of particular importance for fractured composites, since an integral equation contains the singularity at the vertex of the fracture a priori.

Generally, the effective properties of random dispersed composite materials can be theoretically determined using statistical correlation functions [27,28,29]. However, the constructive study is limited to low-order correlation functions. As an alternative, this paper continues the development of a computationally efficient methodology based on structural sums, extending the framework of [1] to elastic problems.

Bergman et al. [30] determined the upper and lower limits for effective conductivity for a periodic case of two-phase composites. The Stieltjes function was exploited to develop a theory of such composites.

Empirical formulas, self-consistent approximations, and their limitations in the theory of composites were discussed in Part I. The main empirical models in the case of elastic composites are presented in [31,32]. In [17,33], these models were determined to be valid up to $O(f^{2})$ and at most up to $O(f^{3})$ for a macroscopically isotropic compound, where *f* denotes the concentration of inclusions. Although various analytical expressions for periodic structures that adhere to homogenization principles have been derived, many of them do not conform to the requirements of asymptotic analysis.

Important results were obtained by Eshelby in [34,35]. The stress–strain field inside an elliptic inclusion embedded in an infinite space is stated to be uniform under the uniform external elastic field applied at infinity. Maxwell’s approach, in combination with Eshelby’s exact equations, yielded analytical formulas for the effective tensor of ellipses up to $O(f^{2})$.

Multiple authors in various settings tried to apply Eshelby’s formulas to determine higher-order terms in the effective tensorial expansion of general two-phase composites. One of the popular tricks was suggested in [36]. It was used in [37] when the eigenstrain in the inclusions was replaced by its average. It is explicitly demonstrated for circular inclusions [33] that the latter approximation holds only up to $O(f^{2})$. However, this approximation was used in [38] to obtain an analytical formula for the effective tensor in the form of a polynomial in *f* in the case of regular arrays of identical circular inclusions. The linear term is correct, while the terms $O(f^{2})$ are erroneous far beyond their precision. A formula valid for a dilute composite cannot be extended to higher concentrations beyond the established precision.

This case, along with numerous others, underscores the persistent gap in the accuracy of asymptotic methodologies applied within the field. Only a limited number of studies adhere to the principles of asymptotic analysis; see [13,39,40,41] and the references therein.

The objective of this paper is to derive analytical expressions for the effective elastic constants of 2D dispersed random composites. The primary theoretical framework designed for such a task is homogenization theory, which is fundamentally based on asymptotic analysis. The theory of stationary fields in elastic composites is based on the fourth-order elliptic PDE [42,43]. This requires a more complicated mathematical model than the model for conducting materials governed by the second-order PDE employed in Part I.

In the present paper, analytical expressions, formulated symbolically in terms of material properties and geometric parameters, have proven instrumental in solving various optimal design problems. In particular, one can address the case of layered composites, frequently considered as a 1D elastic problem because of their geometry. Indeed, the analytical formulas derived were successfully applied to layered structures [44,45]. Similar analytical tools have found broad applications in diverse fields such as aerospace engineering [46], marine sciences, biomaterials [47], medicine [48], etc. Further development along such lines to spatially dispersed composites promises to be useful for graphene-based composites [49], for enhanced printing performance [50], and for cast aluminum composites [22,23], among others.

A theoretical challenge to be addressed in the present paper is the identification of conditions under which Hill’s self-consistent approach aligns with the principles of mathematical homogenization. These conditions are explicitly formulated and validated in the present paper. This framework enables the application of the theory of the analytic Representative Volume Element (*a*RVE) proposed in [51] and summarized in [1].

## 2. Dispersed Random Composite

Consider the case of elastic inclusions $D_{m}$ (m=1,2,…), with Poisson’s ratio $\nu_{m}$ and shear modulus $\mu_{m}$, embedded within a matrix *D*, characterized by elastic constants ν and μ. The sets *D* or the union set $\cup_{m=1}^{\infty }D_{m}$ must be measurable [5,52]. The latter property ensures proper application of the homogenization methods.

Spatial or geometric randomness is simulated by the probabilistic distribution of inclusions $D_{m}$ in space. Such a probabilistic description becomes more transparent if we consider a simplified case of 2D two-phase composites with identical non-overlapping circular inclusions $D_{m}=\{z\in C:\;|z-a_{m}\left|\;<\;r\right\}$ (m=1,2,…). Here, the complex coordinate $z=x_{1}+ix_{2}$ is introduced in the plane $R^{2}\equiv C$, and $a_{m}$ denotes the center of the disk $D_{m}$ of radius *r*. The conditions prohibiting overlaps imply the inequality $|a_{m}-a_{k}|\geq 2r$ for m≠k. In this case, in the complex plane, we arrive at an infinite set of centers that fill the whole plane

(1) $$A=\{a_{1},a_{2},a_{3},\ldots \}.$$

The homogenization theory was developed for sets A that satisfy the property of stationarity in the strict sense [5]. The latter property ensures that the probability distribution of any set of samples of A is invariant with respect to spatial translations. In particular, this implies that the concentration of A defined as the number of points per unit area is correctly defined throughout the plane. The existence of concentration over the whole space for a layered 1D composite is sufficient for the existence of effective constants.

The stationarity of A means that the probability distribution of any set of samples of A does not vary with any translation. Based on the ergodicity hypothesis [5,12,52], a typical element of the considered random set can be selected to compute the effective constants. Therefore, an infinite typical set may represent a class of random composites for almost all probabilistic events.

Naturally, this leads to the following question. Is it always possible to determine a finite typical set? The answer is negative. As noted on Page 41 of [53], the crystal syngonies (classes of symmetric composites) are not associated with ideal macroscopically isotropic composites. In other words, a triply periodic, dispersed, macroscopically isotropic elastic composite cannot exist except in cases where the component materials possess special elastic constants. The same limitation applies to macroscopically isotropic suspensions composed of hard spherical particles. However, this constraint does not extend to conductive composites. For example, a regular cubic array of spheres constitutes a macroscopically isotropic conducting composite. In contrast, for two-dimensional composites, isotropic crystal syngonies can be constructed with relative ease [33].

In addition to the above theoretical question, the approximate isotropy is important in practice. To deal with a finite number of inclusions, random composites are considered below as periodic stochastic structures [1]. We introduce a fundamental domain *Q* generated by two fundamental translation vectors $\omega_{j}$ (j=1,2) expressed by complex numbers.

**Figure 1:**
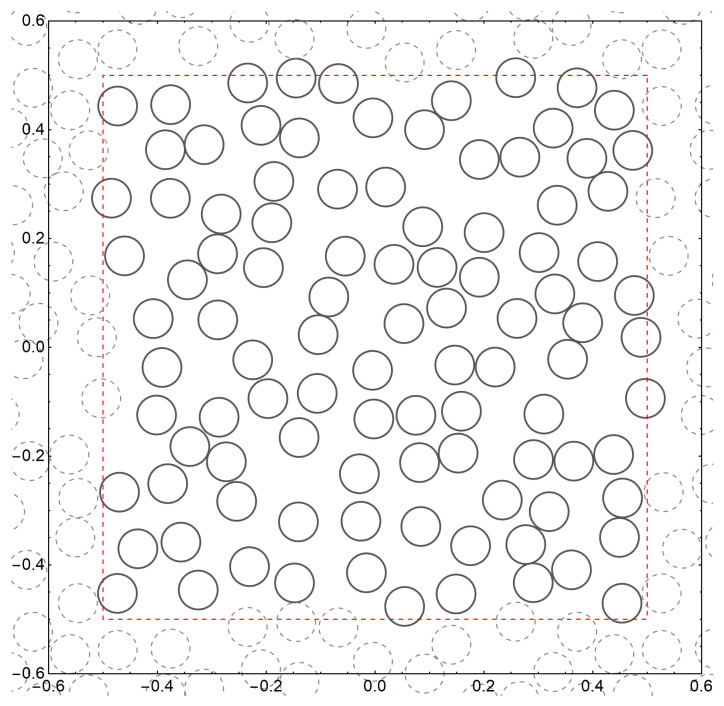
Double-periodic square cell shown with the red dashed lines. The dashed circles show disks that are periodically translated copies of the disks drawn with solid lines.

The unit square domain $Q\equiv Q_{0,0}$ is shown in Figure 1 for which $\omega_{1}=1$ and $\omega_{2}=i$. The translated domains $Q_{m_{1},m_{2}}=Q+m_{1}+im_{2}$ for $m_{1},m_{2}=0\pm 1,\pm 2,\ldots$) periodically fill out the whole plane. The set A is reduced by translations to the finite set

(2) $$A_{N}=\left\{a_{1},a_{2},\ldots ,a_{N}\right\},$$

where *N* stands for the number of inclusions per periodicity cell. Then, we arrive at the following second theoretical question. Consider a set of finite domains $Q_{N}$ that contains the sets (2). Let the boundary $\partial Q_{N}$ extend to infinity as N→∞. Assume that the composite is macroscopically isotropic. Let us introduce the ratio of the average shear modulus $\mu_{N}$ to the shear modulus of the host μ expressed by the average stress components on $Q_{N}$ [33]

(3) $$\frac{\mu_{N}}{\mu }=\frac{{\langle \sigma_{12}\rangle }_{N}}{2{\langle \epsilon_{12}\rangle }_{N}}.$$

Does the limit

(4) $$\lim_{\partial Q_{N}\to \infty }\mu_{N}=\mu_{e}$$

exists and tends to the effective shear modulus? The positive answer to this question could allow us to consider the domain $Q_{N}$ for sufficiently large *N* and approximately compute $\mu_{e}$ by (3). Some authors tacitly assume the existence of the limit (4) and deploy a self-consistent method referred to as Hill’s method [14,15]. However, the limit (4) depends on the exterior shape $\partial Q_{N}$ tending to infinity. This leads to the failure of some popular computational methods and a revision of Hill’s conception discussed in Section 4.

The constructive investigation of the limit (4) may be performed by the representation of the effective constants in terms of the contrast expansions, which lead to conditionally convergent series [12,33]. This effect was first noted by Rayleigh [6], who demonstrated that an extension of a finite set of disks to an infinite regular array leads to a conditionally convergent series in the formula for its effective conductivity. This effect was explained in [54,55] in terms of an additional polarization charge of the external surface. The emergence of a conditionally convergent series was also observed and explained in [56] for 2D elastic composites. Relevant mathematical details can be found in the articles and works cited in [12].

We now summarize the computational consequences of conditional convergence. Consider a doubly periodic domain *Q* and a bounded fragment $Q_{N}$ of the plane. Figure 1 illustrates this geometry, where the dashed red curve bounds $Q_{N}$.

Let the stress tensors $\sigma_{ij}^{\left(N\right)}\left(x\right)$ be found from a boundary value problem stated in a finite domain $Q_{N}$ and $\sigma_{ij}\left(x\right)$ be found from a periodicity problem. Relate these tensors by equation $\sigma_{ij}\left(x\right)=\sigma_{ij}^{\left(N\right)}\left(x\right)+\Delta \sigma_{ij}^{\left(N\right)}\left(x\right)$, $x\in Q_{N}$. The local field $\sigma_{ij}^{\left(N\right)}\left(x\right)$ describes the stresses in $Q_{N}$, near its boundary and far from the boundary, say, in $Q_{N}^{\prime }\subset Q_{N}$. The difference $\Delta \sigma_{ij}^{\left(N\right)}\left(x\right)$ for $x\in Q_{N}^{\prime }$ is insignificant. Calculate the averaged fields $\sigma_{ij}^{\left(N\right)}$ over $Q_{N}$ and $\sigma_{ij}$ over *Q* and find the averaged elastic constants

(5) $$\mu_{e}=\mu_{N}\left(\partial Q_{N}\right)+\delta \left(\partial Q_{N}\right)\;\Delta \mu_{N}\left(\partial Q_{N}\right),$$

where $\mu_{e}$ is a constant for the homogenized medium and its terms $\mu_{N}\left(\partial Q_{N}\right)$, $\Delta \mu_{N}\left(\partial Q_{N}\right)$ depend not only on *N*, but also on the shape of the boundary $\partial Q_{N}$. Here, the McPhedran’s shape factor $\delta (\partial Q_{N})$, depending only on the shape, is introduced. The shape is not determined by the size of the domain. For instance, rectangular, circular, elliptical, etc., denote the classes of shapes independent of the size. Every class contains similar domains, i.e., {αV} for a fixed domain *V* and all positive numbers α; rotations of *V* are excluded. In [6,54,55] it was established that $\delta (\partial Q_{N})$ vanishes for a rectangular shape and does not equal zero up to $O(f^{3})$ for almost all other shapes.

For simplicity, let us fix the shape *L* of $\partial Q_{N}$ and find the limit form of Equation (5) as *N* tends to infinity

(6) $$\mu_{e}=\mu_{L}+\delta \left(L\right)\;c\mu_{L},$$

where the term $\Delta \mu_{L}$ is of the order $O(f^{2})$. It follows from (6) that the computation of $\mu_{N}\left(\partial Q_{N}\right)$ for a sufficiently large *N* is sensible if only the shape factor δ(L) vanishes. If not, the constant $\mu_{L}$ yields the required constant $\mu_{e}$ only up to $O(f^{2})$. Explicit examples of the discrepancy between $\mu_{L}$ and $\mu_{e}$ are given in [17]. The differential and Mori–Tanaka scheme can be considered as an extension of $\partial Q_{N}$ to infinity by incremental addition to the concentration or simply increasing *N*. Hence, they yield effective constants only up to $O(f^{2})$ [13,17].

In summary, we can say that the computation of local fields in a finite sample by solving a boundary value problem is one of the main problems in the theory of composites. The computation of the effective constants is another problem for which the impact of the boundary of order $O(f^{2})$ must be excluded. It can be accomplished in the framework of homogenization by considering the periodic problems of Section 3 or of the problems for finite domains with zero shape factor, δ(L)=0, considered in Section 4.

## 3. Elastic Problems for a Doubly Periodic Composite

Consider a composite consisting of circular elastic inclusions embedded in the elastic matrix without overlaps. This approach can also be extended to multiphase dispersed composites with inclusions of varying shapes. Guided by the principles of homogenization theory [1], we consider a double periodic array consisting of circular inclusions, as shown in Figure 1.

To be more precise, let the domains *D* and $D_{m}$ be occupied by elastic materials with shear moduli μ and $\mu_{m}$ and with Poisson’s ratios ν and $\nu_{m}$, respectively. One can also introduce the so-called Muskhelishvili constant κ=3−4ν [42] for plane strain. The bulk modulus $k=\frac{\mu }{1-2\nu }=\frac{2\mu }{\kappa -1}$ can also be useful. The corresponding elastic constants for inclusions are denoted by $\kappa_{m}$ and $k_{m}$ (m=1,2,…). It is assumed that the contact between the components is perfect.

The components of the stress tensor can be determined from the Kolosov–Muskhelishvili formulas [42]

(7) σ11+σ22=4Re φm′(z),z∈Dm,4Re φ0′(z), z∈D,

$$\sigma_{11}-\sigma_{22}+2i\sigma_{12}=\left\{\begin{array}{cc} -2\left[z\bar{\varphi_{m}^{\prime \prime }\left(z\right)}+\bar{\psi_{m}^{\prime }\left(z\right)}\right], & z\in D_{m},\\ -2\left[z\bar{\varphi_{0}^{\prime \prime }\left(z\right)}+\bar{\psi_{0}^{\prime }\left(z\right)}\right], & \;z\in D, \end{array}\right.$$

where the functions $\varphi_{0}\left(z\right)$ and $\psi_{0}\left(z\right)$ are analytical in *D* and satisfy the periodicity conditions discussed below. The functions $\varphi_{m}\left(z\right)$ and $\psi_{m}\left(z\right)$ are analytical in $D_{m}$. They are also differentiable twice in the closures of the considered domains.

Analogous formulas hold for the strain tensor components [42]

(8) ϵ11+ϵ22=κm−1μmRe φm′(z),z∈Dm,κ−1μRe φ0′(z), z∈D,

$$\epsilon_{11}-\epsilon_{22}+2i\epsilon_{12}=\left\{\begin{array}{cc} -\frac{1}{\mu_{m}}\left[z\bar{\varphi_{m}^{\prime \prime }\left(z\right)}+\bar{\psi_{m}^{\prime }\left(z\right)}\right], & z\in D_{m},\\ -\frac{1}{\mu }\left[z\bar{\varphi_{0}^{\prime \prime }\left(z\right)}+\bar{\psi_{0}^{\prime }\left(z\right)}\right], & \;z\in D. \end{array}\right.$$

Let $u=(u_{1},u_{2})$ and $u_{m}$ stand for displacements in the matrix and within inclusions, respectively. They are related to the components of the strain tensor by the equations

(9) $$\epsilon_{ij}=\frac{1}{2}\left(\frac{\partial u_{i}}{\partial x_{j}}+\frac{\partial u_{j}}{\partial x_{i}}\right),\;i,j=1,2,$$

written in *D*.

The displacement vectors can be written in terms of complex potentials. For example, we have [42]

(10) $$u\left(z\right)=\frac{1}{2\mu }\left(\kappa \varphi_{0}\left(z\right)-z\bar{\varphi_{0}^{\prime }\left(z\right)}-\bar{\psi_{0}\left(z\right)}\right),\;z\in D,$$

up to an additive constant.

Consider for definiteness the square unit cell $Q_{0,0}$ defined by two fundamental translation vectors expressed by the complex numbers $\omega_{1}=1$ and $\omega_{2}=i$. The area of $Q_{0,0}$ is equal to unity. Introduce cells

(11) $$Q_{\left(p,q\right)}:=Q_{\left(0,0\right)}+p+iq=\left\{z\in C:z-\left(p+iq\right)\in Q_{\left(0,0\right)}\right\},$$

for integers *p* and *q*.

Introduce the complex coordinate for the center of the domain $D_{m}$

(12) $$a_{m}=\frac{1}{|D_{m}|}\int_{D_{m}}\left(x_{1}+ix_{2}\right)dx_{1}dx_{2}.$$

It is assumed that $a_{m}$(m=1,2,…,N) belongs to the center cell $Q_{0,0}$. Therefore, we have *N* inclusions per unit square, translated to the plane with translation vectors $a_{m}+p+iq$(m=1,2,…,N) for integers *p* and *q*.

The perfect contact between the components of the medium means the continuity of traction and displacement across the boundary of the components. Let $\partial D_{m}$ be a smooth curve oriented in the positive direction with the unit outward normal vector $n=(n_{1},n_{2})$. Let the limit boundary values of the local fields be denoted by superscripts + and −, so that $u_{i}^{+}\left(t\right):=\lim_{z\to t\in D_{m}}u_{i}\left(z\right)$ and $u_{i}^{-}\left(t\right):=\lim_{z\to t\in D}u_{i}\left(z\right)$. Then, the condition of perfect contact can be written in the form

(13) $$\sigma_{ij}^{+}n_{j}\left(t\right)=\sigma_{ij}^{-}n_{j}\left(t\right),\;u_{i}^{+}\left(t\right)=u_{i}^{-}\left(t\right),\;t\in \partial D_{m}\;\left(m=1,2,\ldots ,N\right),$$

where the Einstein summation over dummy indices is used.

The relations (13) in terms of complex potentials become [42]

(14) $$\begin{array}{c} \varphi_{m}\left(t\right)+t\bar{\varphi_{m}^{\prime }\left(t\right)}+\bar{\psi_{m}\left(t\right)}=\varphi_{0}\left(t\right)+t\bar{\varphi_{0}^{\prime }\left(t\right)}+\bar{\psi_{0}\left(t\right)}+C_{m}, \end{array}$$

(15) $$\begin{array}{c} \kappa_{m}\varphi_{m}\left(t\right)-t\bar{\varphi_{m}^{\prime }\left(t\right)}-\bar{\psi_{m}\left(t\right)}=\frac{\mu_{m}}{\mu }\left(\kappa \varphi_{0}\left(t\right)-t\bar{\varphi_{0}^{\prime }\left(t\right)}-\bar{\psi_{0}\left(t\right)}\right),\\ t\in \partial D_{m}\;\left(m=1,2,\ldots ,N\right), \end{array}$$

where $C_{m}$ are undetermined constants. Equation (14) shows the equality of forces from different sides of $\partial D_{m}$. Equation () expresses the equality of displacements. Let $D=Q_{0,0}\setminus \cup_{m=1}^{N}\left(D_{m}\cup \partial D_{m}\right)$ denote the complement to all closed domains $D_{m}\cup \partial D_{m}$ to the fundamental domain $Q_{0,0}$. Let the centers $a_{m}$ belong to the central cell $Q_{0,0}$.

The functions $\varphi_{0}\left(z\right)$ and $\psi_{0}\left(z\right)$ are represented in the form

(16) $$\varphi_{0}\left(z\right)=bz+\varphi \left(z\right),\;\psi_{0}\left(z\right)=\Gamma z+\psi \left(z\right).$$

where φ(z) and ψ(z) are analytic in *D*. They are also continuously differentiable twice in the closure of *D*. The real constant *b* and complex constant Γ are given. It follows from [25] that φ(z) is quasi-periodic, so that

(17) $${\left[\varphi \left(z\right)\right]}_{j}:=\varphi \left(z+\omega_{j}\right)-\varphi \left(z\right)=\xi_{j}\omega_{j}\;\left(j=1,2\right)$$

for some constants $\xi_{j}$. The function ψ(z) satisfies the quasi-periodicity conditions [25]

(18) $${\left[\psi \left(z\right)\right]}_{j}:=\psi \left(z+\omega_{j}\right)-\psi \left(z\right)=-\bar{\omega_{j}}\varphi^{\prime }\left(z\right)+\eta_{j}\omega_{j}\;\left(j=1,2\right)$$

for some constants $\eta_{j}$. These conditions (17) and (18) formulated for complex potentials ensure the periodicity of stresses. Displacements under such conditions appear to be quasi-periodic [25].

The doubly periodic problem was first stated by Natanzon in [7] for a regular hexagonal array of cylinders and developed by Filshtinsky in [8,9,57]. The slight difference between the statements is that the constants *b* and Γ in our approach are given. However, they have to be found when the Natanzon–Filshtinsky method is applied. To properly apply the Natanzon–Filshtinsky technique, it is necessary to fix in advance an average stress tensor $\langle \sigma_{ij}\rangle$ or an average strain tensor $\langle \epsilon_{ij}\rangle$. The Natanzon–Filshtinsky statement complies with the homogenization theory summarized by the following rules or principles.

### Principles of Homogenization

- The boundary value problem is stated for a doubly periodic domain $Q\equiv Q_{0,0}$.
- The conditions of quasi-periodicity (17) and (18) correspond to the external stress tensor to be fixed at infinity. Alternatively, the strain tensor can be posed at infinity.
- The effective elastic constants in macroscopically isotropic composites are determined by averaging the local fields over *Q*.

In part I, it was shown that the antiplane elasticity problem can be considered for finite rectangular cells *Q*, and not necessarily in the periodic statement. This follows from the periodization of initially bounded domains. The analogous question for elastic problems is discussed in the next section.

## 4. Hill’s Conception

**Figure 2:**
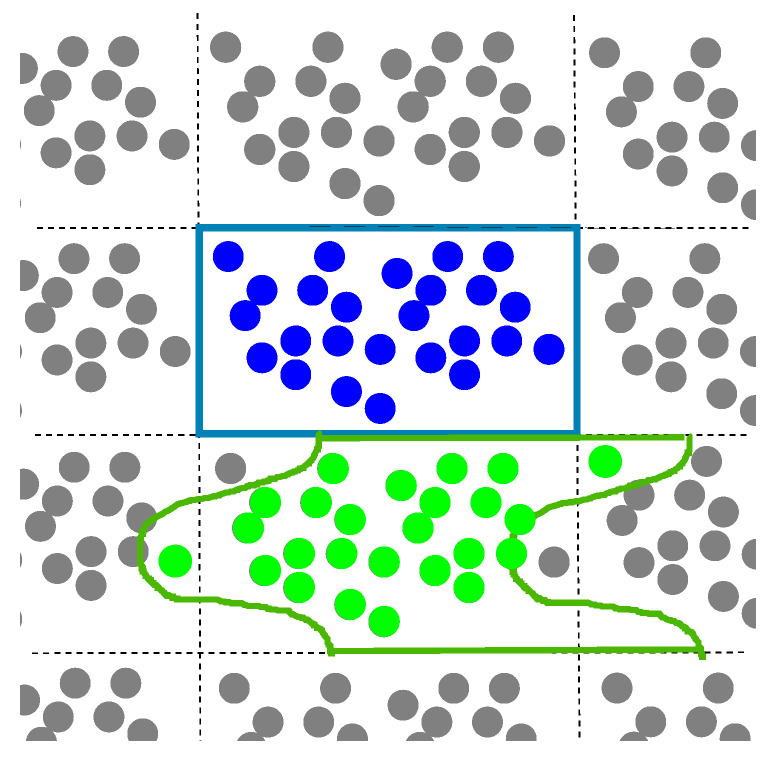
The standard fundamental domain $Q=Q_{\left(0,0\right)}$, a parallelogram (rectangular), is shown in blue. A fundamental domain of another shape is shown in green.

**Figure 3:**
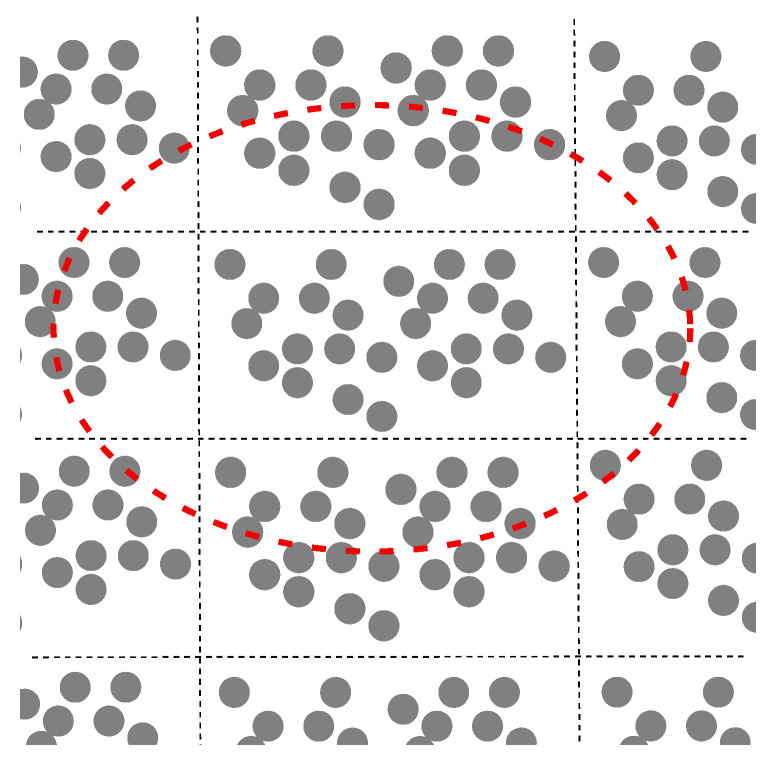
The domain *V* bounded by the red curve is not a fundamental domain of the considered doubly periodic structure.

Hill’s conception [15], closely related to Maxwell’s approach, was developed in 1965 for elastic composites. We now proceed to discuss this approach according to [14,15,16]. The designation *V* is used for a plane domain instead of the fundamental domain *Q*. A fundamental domain periodically tiles the entire plane, as shown in Figure 2. The domain *Q* and a domain of different shapes shown in Figure 2 are the fundamental domains of the same two-dimensional torus constructed by two translation vectors $\omega_{1}$ and $\omega_{2}$. Figure 3 displays a domain *V* that is not fundamental, in contrast to the fundamental domains shown in Figure 2.

Hill showed that the local elastic fields near the boundary ∂V of the domain *V* were equivalent to their averages if energy were considered. Let $u_{i}$ denote the components of displacement; then,

(19) $$\int_{\partial V}\left(u_{i}-\langle \epsilon_{ij}\rangle x_{j}\right)\left(\sigma_{il}-\langle \sigma_{il}\rangle \right)n_{l}ds=0.$$

Hill’s understanding of RVE is explained in the Introduction. Based on relation (19), Hill proposed to consider the following two equivalent boundary value problems for the domain *V*,

1. Linear displacement:
  (20) $$u_{i}=\langle \epsilon_{ij}\rangle x_{j}\;\left(i,j=1,2\right)\;on\;\partial V.$$
2. Uniform traction:
  (21) $$\sigma_{il}n_{l}=\langle \sigma_{il}\rangle n_{l}\;\left(i,l=1,2\right)\;on\;\partial V.$$

The values $\langle \epsilon_{ij}\rangle$ and $\langle \sigma_{il}\rangle n_{l}$ for averaged constants are also given.

Here, the local stress tensor σ(x) is defined by the components $\sigma_{il}\left(x\right)$. The local strain tensor ϵ(x) is defined by $\epsilon_{ij}\left(x\right)$ when x∈V. Let the local elasticity tensor be denoted by C(x). Suppose that the compliance tensor $C_{e}^{-1}$ can be determined after solving the boundary value problem (20) by means of the relation

(22) $$C_{e}^{-1}:\langle \sigma \left(x\right)\rangle =\langle \epsilon \rangle =\langle C^{-1}\left(x\right):\sigma \left(x\right)\rangle ,$$

where 〈ϵ〉 is a given constant tensor.

The effective elasticity tensor $C_{e}$, inverse to the compliance tensor, can be determined by solving the boundary value problem (21). It is determined from the following equation:

(23) $$C_{e}:\langle \epsilon \left(x\right)\rangle =\langle \sigma \rangle =\langle C\left(x\right):\epsilon \left(x\right)\rangle ,$$

where 〈σ〉 is a given constant tensor.

Hill’s approach was not theoretically justified [58], that is, its consistency with the principles of homogenization of Section 3 was not established. It was only confirmed in some cases by means of numerical computations. It was explicitly demonstrated in [17,33] that this approach to the domain *V* shown in Figure 3 was incorrect. The latter demonstration leads to a revision of the naive approach to stochasticity prevalent in the engineering literature on random composites. We argue that it must be corrected by replacing an arbitrarily chosen domain *V* by a fundamental cell *Q*. Then, Hill’s approach becomes consistent with the mathematical homogenization theory. A further step consists of a rigorous constructive definition of RVE, which was proposed in [51] and summarized in [1,12].

Fortunately, in most previous studies, only evident periodic cases of the square and cubic shapes of *V* are considered, tacitly assuming that *V* coincides with the fundamental domain *Q*. However, despite the compelling arguments presented in [59], the oversimplified self-consistent approach remains unreasonably propagated in the literature.

## 5. Asymptotic Formulas for the Effective Elastic Constants

For simplicity, we perform simulations for identical circular inclusions $D_{m}=\{z\in C:\;|z-a_{m}\left|<r\right\}$(m=1,2,…,N), as illustrated in Figure 1 with N=100 disks per unit square cell. In the case of two-phase composites, we set $\mu_{m}=\mu_{1}$ and $\nu_{m}=\nu_{1}$ for all m=1,2,…,N. The concentration of inclusions

(24) $$f=N\pi r^{2},$$

is conventional.

Let us introduce the elastic contrast parameters using Muskhelishvili’s constant $\kappa =\frac{3-\nu }{1+\nu }$ [33],

(25) $$\varrho_{1}=\frac{\frac{\mu_{1}}{\mu }-1}{\frac{\mu_{1}}{\mu }+\kappa_{1}},\;\varrho_{2}=\frac{\kappa \frac{\mu_{1}}{\mu }-\kappa_{1}}{\kappa \frac{\mu_{1}}{\mu }+1},\;\varrho_{3}=\frac{\frac{\mu_{1}}{\mu }-1}{\kappa \frac{\mu_{1}}{\mu }+1}$$

related by the following identity

(26) $$\varrho_{1}=\frac{\varrho_{3}}{1-\varrho_{2}+\varrho_{3}}.$$

The denominator of (26) is equal to the value $\frac{\frac{\mu_{1}}{\mu }+\kappa_{1}}{\frac{\mu_{1}}{\mu }\kappa +1}$, which is always positive.

The following are the definitions and formulas of Appendix A are used. The following structural sum can be written for square arrays,

(27) $$e_{3}^{\left(1)(1\right)}:=\frac{1}{N^{2}}\sum_{k,m=1}^{N}\bar{E_{3}^{\left(1\right)}\left(a_{k}-a_{m}\right)},$$

where $E_{3}^{\left(1\right)}\left(a_{k}-a_{m}\right):=0$ if $a_{k}=a_{m}$.

The torus topology implies that the complex values $\left(a_{k}-a_{m}\right)\equiv {\left(a_{k}-a_{m}\right)}_{tor}$ define the distance between $a_{k}$ and $a_{m}$ so that

(28) $$∥a_{k}-a_{m}∥:=\min_{s_{1},s_{2}=-1,0,1}\left|a_{k}-a_{m}+s_{1}+is_{2}\right|.$$

In [56] it was shown that for a macroscopically isotropic composite material, $e_{3}^{\left(1)(1\right)}=0$. This formula may be used to define the value

(29) $$S_{3}^{\left(1\right)}=-\frac{1}{N^{2}}\sum_{k,m=1}^{N}{\left(a_{k}-a_{m}\right)}_{tor}\bar{E_{3}\left(a_{k}-a_{m}\right)}-\frac{1}{2N^{2}}\sum_{k,m=1}^{N}{℘}_{1}^{\prime }\left(a_{k}-a_{m}\right),$$

where $E_{3}\left(a_{k}-a_{m}\right):=0$ and ${℘}_{1}^{\prime }\left(a_{k}-a_{m}\right):=0$ if $a_{k}=a_{m}$. $S_{3}^{\left(1\right)}$ for the unit square array is found as

(30) $$S_{3}^{\left(1\right)}=\frac{\Gamma^{8}\left(\frac{1}{4}\right)}{384\pi^{3}}\approx 2.50765.$$

Therefore, the function $E_{3}^{\left(1\right)}\left(z\right)$ for the square cell $Q_{0,0}$ must be calculated from Formula (A7) with $S_{3}^{\left(1\right)}$ defined by (30).

For macroscopically isotropic 2D elastic composites made from identical circular inclusions, the effective shear modulus can be calculated using the following formula (4.21) from [33],

(31) μeμ=1+Re ∑s=1∞A(s)fs1−κRe ∑s=1∞A(s)fs

with the coefficients $A^{\left(s\right)}$ written exactly below. Formula (31) was derived from the formula

(32) $$\frac{\mu_{e}}{\mu }=\frac{\langle \sigma_{12}\rangle }{2\langle \epsilon_{12}\rangle },$$

where the averaged components $\langle \sigma_{12}\rangle$ and $\langle \epsilon_{12}\rangle$ were calculated by (7) and (8). The corresponding analytical functions were found from the boundary value problem (14) with B=0 and Γ=i in (16). The method of functional equations [33] was applied.

Let C denote the complex conjugation operator. Consider a natural number *p*, and let *j* be 0 and 1, s=1,2,…,p, $k_{s}=1,2,\ldots ,N$, $\alpha =\sum_{s=1}^{p}\left(n_{s}-j_{s}\right)$, and $n_{s}=2,3,\ldots$. Following [33] (Chapter 4), let us introduce the multi-order elastic structural sums $p=(n_{1},\ldots ,n_{p})$

(33) $$e_{n_{1},\ldots ,n_{p}}^{\left(j_{1},\ldots ,j_{p}\right)\left(l_{1},\ldots ,l_{p}\right)}=\frac{1}{N^{\frac{\alpha }{2}+1}}\sum_{k_{0},k_{1},\ldots ,k_{p}}\prod_{s=1}^{p}C^{l_{s}}E_{n_{s}}^{\left(j_{s}\right)}\left(a_{k_{s-1}}-a_{k_{s}}\right).$$

Several coefficients were explicitly written in [33]. In particular,

(34) $$A^{\left(1\right)}=\varrho_{3},\;A^{\left(2\right)}=-\frac{2}{\pi }\varrho_{3}^{2}e_{3}^{\left(1)(1\right)}=0.$$

The coefficients in (34) depend on the contrast parameter $\varrho_{3}$. Choose p=1, $j_{1}=1$, $l_{1}=1$, and $n_{1}=2$. This gives α=2.

The next coefficients $A^{\left(l\right)}$ were calculated in symbolic form in [33]. The coefficients depend on all the contrast parameters for l≥3. Thus,

(35) $$A^{\left(3\right)}=\frac{1}{\pi^{2}}\varrho_{3}\left[4\varrho_{3}^{2}e_{3,3}^{\left(1,1)(1,0\right)}+6\varrho_{3}e_{4}^{\left(0)(1\right)}+\frac{\varrho_{1}-\varrho_{3}}{1+\varrho_{1}}\left(e_{2,2}^{\left(0,0)(1,0\right)}-e_{2,2}^{\left(0,0)(1,1\right)}\right)\right].$$

In order to calculate $A^{\left(3\right)}$, let us write the structural sums in their extended form. Let p=2, $j_{1}=j_{2}=0$, $l_{1}=1$, $l_{2}=0$, and $n_{1}=n_{2}=2$. Then, α=4 and

(36) $$e_{2,2}^{\left(0,0)(1,0\right)}=\frac{1}{N^{3}}\sum_{k_{0},k_{1},k_{2}=1}^{N}\bar{E_{2}\left(a_{k_{0}}-a_{k_{1}}\right)}E_{2}\left(a_{k_{1}}-a_{k_{2}}\right)\equiv e_{2,2},$$

since $e_{2,2}\geq 0$ [60]. Here, $S_{2}$ from (A9) is defined as zero.

Let p=2, $j_{1}=j_{2}=0$, $l_{1}=l_{2}=1$, and $n_{1}=n_{2}=2$. Then, α=4 and

(37) $$e_{2,2}^{\left(0,0)(1,1\right)}=\frac{1}{N^{3}}\sum_{k_{0},k_{1},k_{2}=1}^{N}\bar{E_{2}\left(a_{k_{0}}-a_{k_{1}}\right)}\bar{E_{2}\left(a_{k_{1}}-a_{k_{2}}\right)}.$$

Then, the expression from (35) becomes

(38) Δe2,2:=e2,2(0,0)(1,0)−e2,2(0,0)(1,1)=2N3∑k0,k1,k2=1NE2(ak0−ak1)¯Im E2(ak1−ak2).

Let p=2, $j_{1}=j_{2}=1$, $l_{1}=1$, $l_{2}=0$, and $n_{1}=n_{2}=3$. Then, α=4 and

(39) $$e_{3,3}^{\left(1,1)(1,0\right)}=\frac{1}{N^{3}}\sum_{k_{0},k_{1},k_{2}=1}^{N}\bar{E_{3}^{\left(1\right)}\left(a_{k_{0}}-a_{k_{1}}\right)}E_{3}^{\left(1\right)}\left(a_{k_{1}}-a_{k_{2}}\right)=-\frac{1}{N^{3}}\sum_{k=1}^{N}{\left|\sum_{m=1}^{N}E_{3}^{\left(1\right)}\left(a_{k}-a_{m}\right)\right|}^{2}.$$

In order to express $e_{4}^{\left(0)(1\right)}$ explicitly, we take p=1, $j_{1}=0$, $l_{1}=1$, and $n_{1}=4$. Then, α=4 and

(40) $$e_{4}^{\left(0)(1\right)}=\frac{1}{N^{3}}\sum_{k_{0},k_{1}}^{N}\bar{E_{4}\left(a_{k_{0}}-a_{k_{1}}\right)}\equiv \bar{e_{4}}.$$

The next coefficients have the form

(41) $$\begin{array}{c} A^{\left(4\right)}=\frac{1}{\pi^{3}}\left[-2\varrho_{1}\varrho_{2}\varrho_{3}e_{3,3}^{\left(0,0)(1,0\right)}-12\varrho_{3}^{3}e_{4,3}^{\left(0,1)(1,0\right)}-12\varrho_{3}^{3}e_{3,4}^{\left(1,0)(1,0\right)}\right.\\ \;\;\;\;\;\;\;\;\;\;\left.-18\varrho_{3}^{3}e_{4,4}^{\left(1,1)(1,0\right)}-8\varrho_{3}^{4}e_{3,3,3}^{\left(1,1,1)(1,0,1\right)}\right.\\ \;\;\;\;\;\;\;\;\;\;\left.-2\varrho_{3}^{2}\frac{\varrho_{3}-\varrho_{1}}{1+\varrho_{1}}\left(e_{2,2,3}^{\left(0,0,1)(0,0,1\right)}+e_{2,2,3}^{\left(0,0,1)(1,1,0\right)}+2e_{2,2,3}^{\left(0,0,1)(1,0,1\right)}\right)\right], \end{array}$$

(42) A(5)=1π43ϱ3(ϱ1ϱ2+12ϱ32)e4,4(0,0)(1,0)+24ϱ335Ree5,4(0,1)(1,0)+2e5,5(1,1)(1,0)+8ϱ1ϱ2ϱ32e3,3,3(0,0,1)(1,0,1)+16ϱ35e3,3,3,3(1,1,1,1)(1,0,1,0)+24ϱ342e4,3,3(0,1,1)(1,0,1)+e3,4,3(1,0,1)(1,0,1)+3e4,4,3(1,1,1)(1,0,1)+12ϱ32ϱ1−ϱ31+ϱ1e2,2,4(0,0,0)(1,0,1)+e2,3,4(0,0,1)(1,0,1)−Ree2,2,4(0,0,0)(1,1,0)+e2,3,4(0,0,1)(1,1,0)+ϱ3ϱ1−ϱ31+ϱ12e2,2,2,2(0,0,0,0)(1,0,1,0)−2e2,2,2,2(0,0,0,0)(1,0,1,1)+e2,2,2,2(0,0,0,0)(1,1,0,0)+4ϱ33ϱ1−ϱ31+ϱ12Ree2,2,3,3(0,0,1,1)(1,0,1,0)−2e2,2,3,3(0,0,1,1)(1,1,0,1)−e3,2,2,3(1,0,0,1)(1,0,0,1)+e3,2,2,3(1,0,0,1)(1,0,1,0)

(43) A(6)=1π5−4ϱ3(ϱ1ϱ2+50ϱ32)e5,5(0,0)(1,0)−ϱ33360Ree6,5(0,1)(1,0)+e6,6(1,1)(1,0)+ϱ32ϱ1−ϱ31+ϱ1−40e2,3,5(0,0,0)(1,0,1)+40Ree2,3,5(0,0,0)(1,1,0)−24e2,4,5(0,0,1)(1,0,1)+24Ree2,4,5(0,0,1)(1,1,0)+ϱ1ϱ2ϱ32−24e3,3,4(0,0,0)(1,0,1)−36e3,4,4(0,0,1)(1,0,1)+12ϱ32(ϱ1ϱ2+12ϱ32)e4,4,3(0,0,1)(1,0,1)+ϱ34−72e4,3,4(0,1,0)(1,0,1)−216e4,4,4(0,1,1)(1,0,1)−240e5,4,3(0,1,1)(1,0,1)−240e3,5,4(1,0,1)(1,0,1)−192e3,5,5(1,1,1)(1,0,1)−216e4,5,4(1,1,1)(1,0,1)−4ϱ1ϱ2ϱ3ϱ1−ϱ31+ϱ1Ree2,2,3,3(0,0,0,0)(1,0,1,0)−e2,2,3,3(0,0,0,0)(1,1,0,1)+12ϱ33ϱ1−ϱ31+ϱ1Re−2e2,2,4,3(0,0,0,1)(1,0,1,0)−2e2,2,3,4(0,0,1,0)(1,0,1,0)−3e2,2,4,4(0,0,1,1)(1,0,1,0)−2e2,3,4,3(0,0,1,1)(1,0,1,0)−2e4,2,2,3(0,0,0,1)(1,0,1,0)−2e3,2,3,4(1,0,0,1)(1,0,1,0)+2e2,2,4,3(0,0,0,1)(1,1,0,1)+2e2,2,3,4(0,0,1,0)(1,1,0,1)+3e2,2,4,4(0,0,1,1)(1,1,0,1)+2e2,3,4,3(0,0,1,1)(1,1,0,1)+2e4,2,2,3(0,0,0,1)(1,0,0,1)+2e3,2,3,4(1,0,0,1)(1,0,0,1)−2ϱ3ϱ1−ϱ31+ϱ12e2,3,3,2(0,0,0,0)(1,0,1,0)+e2,3,3,2(0,0,0,0)(1,1,0,0)−e2,3,3,2(0,0,0,0)(1,1,0,1)−8ϱ1ϱ2ϱ332Ree3,3,3,3(0,0,1,1)(1,0,1,0)+e3,3,3,3(1,0,0,1)(1,0,1,0)−24ϱ354e4,3,3,3(0,1,1,1)(1,0,1,0)+3e3,4,4,3(1,1,1,1)(1,0,1,0)+Re4e3,4,3,3(1,0,1,1)(1,0,1,0)+6e3,3,4,4(1,1,1,1)(1,0,1,0)+2ϱ32ϱ1−ϱ31+ϱ122Ree2,2,2,2,3(0,0,0,0,1)(1,0,1,1,0)+e2,2,3,2,2(0,0,1,0,0)(1,0,1,0,0)+e2,2,2,2,3(0,0,0,0,1)(1,1,0,1,0)−2e2,2,2,2,3(0,0,0,0,1)(1,0,1,0,1)−e2,2,3,2,2(0,0,1,0,0)(1,0,1,0,1)−e2,2,3,2,2(0,0,1,0,0)(1,1,0,1,1)−e3,2,2,2,2(1,0,0,0,0)(1,0,0,1,1)+16ϱ34ϱ1−ϱ31+ϱ1Ree2,2,3,3,3(0,0,1,1,1)(1,1,0,1,0)+e3,2,2,3,3(1,0,0,1,1)(1,0,0,1,0)−e3,2,2,3,3(1,0,0,1,1)(1,0,1,0,1)−e3,3,3,2,2(1,1,1,0,0)(1,0,1,0,1)−32ϱ36e3,3,3,3,3(1,1,1,1,1)(1,0,1,0,1).

The effective bulk modulus for the regular hexagonal array of disks was expressed as an approximate formula given by Equation (3.75) from [33].

Following the method of Chapter 4 from [33], we extend it to the effective bulk modulus for macroscopically isotropic 2D elastic composites made of identical disks

(44) kek=1−f+Re ∑s=1∞B(s)+C(s)fs1−f+Re ∑s=1∞B(s)+kk1C(s)fs,

with the coefficients $B^{\left(s\right)}$ and $C^{\left(s\right)}$ written exactly below. Formula (44) was derived from the formula

(45) $$\frac{k_{e}}{k}=\frac{\langle \sigma_{11}+\sigma_{22}\rangle }{2\langle \epsilon_{11}+\epsilon_{22}\rangle },$$

where the averaged terms were calculated by (7) and (8). By means of symbolic computations with $Mathematica^{®}$, several low-order coefficients can be found,

(46) $$B^{\left(1\right)}=B^{\left(2\right)}=0,\;\;B^{\left(3\right)}=\frac{2}{\pi^{2}}\frac{\varrho_{3}-\varrho_{1}}{1+\varrho_{1}}e_{2,2}^{\left(0,0)(0,1\right)},$$

(47) $$B^{\left(4\right)}=\frac{4}{\pi^{3}}\frac{\varrho_{3}-\varrho_{1}}{1+\varrho_{1}}\left(\varrho_{3}e_{2,3,2}^{\left(0,1,0)(0,1,0\right)}-e_{3,3}^{\left(0,0)(0,1\right)}\right),$$

(48) $$\begin{array}{c} B^{\left(5\right)}=\frac{2}{\pi^{4}}\frac{\varrho_{3}-\varrho_{1}}{{\left(1+\varrho_{1}\right)}^{2}}\left(3\left(1+\varrho_{1}\right)e_{4,4}^{\left(0,0)(0,1\right)}-6\left(1+\varrho_{1}\right)\varrho_{3}e_{2,4,2}^{\left(0,0,0)(0,1,0\right)}-\right.\\ 6\varrho_{3}\left(1+\varrho_{1}\right)e_{2,4,3}^{\left(0,1,0)(0,1,0\right)}-6\varrho_{3}\left(1+\varrho_{1}\right)e_{3,4,2}^{\left(0,1,0)(0,1,0\right)}+\left(\varrho_{1}-\varrho_{3}\right)e_{2,2,2,2}^{\left(0,0,0,0)(0,1,0,1\right)}+\\ \left.\left(\varrho_{1}-\varrho_{3}\right)e_{2,2,2,2}^{\left(0,0,0,0)(0,1,1,0\right)}+4\varrho_{3}^{2}\left(1+\varrho_{1}\right)e_{2,3,3,2}^{\left(0,1,1,0)(0,1,0,1\right)}\right), \end{array}$$

(49) $$\begin{array}{c} B^{\left(6\right)}=\frac{4}{\pi^{5}}\frac{\varrho_{1}-\varrho_{3}}{{\left(1+\varrho_{1}\right)}^{2}}\left(2\left(1+\varrho_{1}\right)e_{5,5}^{\left(0,0)(0,1\right)}-10\left(1+\varrho_{1}\right)\varrho_{3}e_{2,5,3}^{\left(0,0,0)(0,1,0\right)}\right.\\ -6\varrho_{3}\left(1+\varrho_{1}\right)e_{2,5,4}^{\left(0,1,0)(0,1,0\right)}-10\varrho_{3}\left(1+\varrho_{1}\right)e_{3,5,2}^{\left(0,0,0)(0,1,0\right)}-12\varrho_{3}\left(1+\varrho_{1}\right)e_{3,5,3}^{\left(0,1,0)(0,1,0\right)}\\ -6\varrho_{3}\left(1+\varrho_{1}\right)e_{4,5,2}^{\left(0,1,0)(0,1,0\right)}+\left(\varrho_{1}-\varrho_{3}\right)e_{2,2,3,3}^{\left(0,0,0,0)(0,1,0,1\right)}+\left(\varrho_{1}-\varrho_{3}\right)e_{2,2,3,3}^{\left(0,0,0,0)(0,1,1,0\right)}\\ +\varrho_{1}\varrho_{2}\left(1+\varrho_{1}\right)e_{2,3,3,2}^{\left(0,0,0,0)(0,1,0,1\right)}+6\varrho_{3}^{2}\left(1+\varrho_{1}\right)e_{2,4,3,2}^{\left(0,0,1,0)(0,1,0,1\right)}+6\varrho_{3}^{2}\left(1+\varrho_{1}\right)e_{2,3,4,2}^{\left(0,1,0,0)(0,1,0,1\right)}\\ +6\varrho_{3}^{2}\left(1+\varrho_{1}\right)e_{2,3,4,3}^{\left(0,1,1,0)(0,1,0,1\right)}+9\varrho_{3}^{2}\left(1+\varrho_{1}\right)e_{2,4,4,2}^{\left(0,1,1,0)(0,1,0,1\right)}+\left(\varrho_{1}-\varrho_{3}\right)e_{3,3,2,2}^{\left(0,0,0,0)(0,1,0,1\right)}\\ +\left(\varrho_{1}-\varrho_{3}\right)e_{3,3,2,2}^{\left(0,0,0,0)(0,1,1,0\right)}+6\varrho_{3}^{2}\left(1+\varrho_{1}\right)e_{3,4,3,2}^{\left(0,1,1,0)(0,1,0,1\right)}+\varrho_{3}\left(\varrho_{3}-\varrho_{1}\right)e_{2,2,2,3,2}^{\left(0,0,0,1,0)(0,1,0,1,0\right)}\\ +\varrho_{3}\left(\varrho_{3}-\varrho_{1}\right)e_{2,2,2,3,2}^{\left(0,0,0,1,0)(0,1,1,0,1\right)}+\varrho_{3}\left(\varrho_{3}-\varrho_{1}\right)e_{2,3,2,2,2}^{\left(0,1,0,0,0)(0,1,0,0,1\right)}\\ +\varrho_{3}\left(\varrho_{3}-\varrho_{1}\right)e_{2,3,2,2,2}^{\left(0,1,0,0,0)(0,1,0,1,0\right)}\left.-4\varrho_{3}^{3}\left(1+\varrho_{1}\right)e_{2,3,3,3,2}^{\left(0,1,1,1,0)(0,1,0,1,0\right)}\right) \end{array}$$

(50) $$C^{\left(1\right)}=\frac{\varrho_{1}\left(1+\varrho_{3}\right)}{\varrho_{3}\left(1+\varrho_{1}\right)},\;C^{\left(2\right)}=0,$$

(51) $$C^{\left(3\right)}=\frac{2}{\pi^{2}}\frac{\varrho_{1}\left(\varrho_{3}-\varrho_{1}\right)\left(1+\varrho_{3}\right)}{\left(1-\varrho_{1}\right){\left(1+\varrho_{1}\right)}^{2}\varrho_{3}}\left(\varrho_{1}e_{2,2}^{\left(0,0)(1,0\right)}-e_{2,2}^{\left(0,0)(0,1\right)}\right)$$

(52) $$\begin{array}{c} C^{\left(4\right)}=\frac{4}{\pi^{3}}\frac{\varrho_{1}\left(\varrho_{3}-\varrho_{1}\right)\left(1+\varrho_{3}\right)}{\left(1-\varrho_{1}\right){\left(1+\varrho_{1}\right)}^{2}\varrho_{3}}\left(e_{3,3}^{\left(0,0)(0,1\right)}-\varrho_{1}e_{3,3}^{\left(0,0)(1,0\right)}-\right.\;\;\;\;\;\;\;\;\;\;\\ \left.\varrho_{3}e_{2,3,2}^{\left(0,1,0)(0,1,0\right)}+\varrho_{1}\varrho_{3}e_{2,3,2}^{\left(0,1,0)(1,0,1\right)}\right) \end{array}$$

(53) $$\begin{array}{c} C^{\left(5\right)}=\frac{2}{\pi^{4}}\frac{\varrho_{1}\left(\varrho_{3}-\varrho_{1}\right)\left(1+\varrho_{3}\right)}{\left(1-\varrho_{1}\right){\left(1+\varrho_{1}\right)}^{3}\varrho_{3}}\left(-3\left(1+\varrho_{1}\right)e_{4,4}^{\left(0,0)(0,1\right)}+3\varrho_{1}\left(1+\varrho_{1}\right)e_{4,4}^{\left(0,0)(1,0\right)}+\right.\\ 6\varrho_{3}\left(1+\varrho_{1}\right)e_{2,4,2}^{\left(0,0,0)(0,1,0\right)}+6\varrho_{3}\left(1+\varrho_{1}\right)e_{2,4,3}^{\left(0,1,0)(0,1,0\right)}-6\varrho_{1}\varrho_{3}\left(1+\varrho_{3}\right)e_{2,4,2}^{\left(0,0,0)(1,0,1\right)}\\ -6\varrho_{1}\varrho_{3}\left(1+\varrho_{1}\right)e_{2,4,3}^{\left(0,1,0)(1,0,1\right)}+6\varrho_{3}\left(1+\varrho_{1}\right)e_{3,4,2}^{\left(0,1,0)(0,1,0\right)}-6\varrho_{1}\varrho_{3}\left(1+\varrho_{1}\right)e_{3,4,2}^{\left(0,1,0)(1,0,1\right)}+\\ \left(\varrho_{3}-\varrho_{1}\right)e_{2,2,2,2}^{\left(0,0,0,0)(0,1,0,1\right)}+\left(\varrho_{3}-\varrho_{1}\right)e_{2,2,2,2}^{\left(0,0,0,0)(0,1,1,0\right)}-4\varrho_{3}^{2}\left(1+\varrho_{1}\right)e_{2,3,3,2}^{\left(0,1,1,0)(0,1,0,1\right)}+\\ \left.\varrho_{1}\left(\varrho_{1}-\varrho_{3}\right)e_{2,2,2,2}^{\left(0,0,0,0)(1,0,0,1\right)}+\varrho_{1}\left(\varrho_{1}-\varrho_{3}\right)e_{2,2,2,2}^{\left(0,0,0,0)(1,0,1,0\right)}+4\varrho_{1}\varrho_{3}^{2}\left(1+\varrho_{1}\right)e_{2,3,3,2}^{\left(0,1,1,0)(1,0,1,0\right)}\right) \end{array}$$

The next coefficients are very long. However, they can be found explicitly if a symbolic algorithm developed in [33] is applied.

There is a sharp increase in the complexity of the coefficients from the order $f^{2}$ and $f^{3}$ to the higher orders. One can compare simple expressions (34), (46) with complicated formulas (35), (47). This observation demonstrates the impact of geometry on the effective constants beginning from the term $f^{3}$ and explains the popularity of the simple but “universal” empirical formulas valid up to $O(f^{3})$. Sometimes, empirical formulas are written with poorly justified long tails of the higher-order terms in *f*; or are even declared to have an exact/closed form. There is a simple way to verify their validity; see Chapter 9 of [13].

If a formal general formula contains a term in $f^{3}$ independent of geometry, it must be discarded. For macroscopically anisotropic composites, the geometry dependence term begins with $f^{2}$ [59].

## 6. Concept of Investigations Following *a*RVE Theory

### 6.1. General Scheme

The strategic approach to the effective conductivity of randomly dispersed composite materials was summarized in [1]. Here, we present only its main tenets, modifying their formulation for 2D elastic composites. The preliminary steps of Image Analysis were described in [23,61,62].

For starters, one should look at the rigorous Hashin–Shtrikman bounds on effective conductivity. Other similar bounds of higher order were devised for random composites [63]. If the bounds are sufficiently tight, one may stop and take the bounds as the ultimate result. In the opposite case of the too broadly defined bounds, a constructive homogenization might be considered.

Then, we have to go through the following steps suggested in [1] and adjusted for elastic problems:

1. Computation of structural sums.
2. Computation of effective elastic constants through structural sums.
3. Application of resummation to truncated power series.

Steps 1 and 2 were described in detail in Part I. Here, we note that another set of structural sums introduced in the previous sections must be used for elasticity problems.

The steps presented above are concerned with the special case of dispersed composites discussed in this paper and can be extended to other elastic composites. Some extensions require essential development of mathematical methods, while others can be performed using standard methods.

First, note that 3D elastic dispersed composites and 3D viscous suspensions must be considered for a triply periodic cell *Q* in $R^{3}$ with the corresponding quasi-periodicity conditions. The problems are divided into deterministic and stochastic ones. The deterministic problem for a dispersed 2D (3D) composite, porous medium, or viscous suspension means that all the physical and geometrical parameters are fixed. In particular, the constants $\mu_{m}$, $\nu_{m}$, μ, ν (m=1,2,…,N) are given numerically. Inclusions $D_{m}$ are exactly described as geometric objects. Thus, if $D_{m}$ is a disk (ball), its center position $a_{m}$ and radius $r_{m}$ must be given as input, as well as the number of inclusions *N* per cell. The practical implementation of pure numerical methods, such as FEM and the integral equation method, was briefly discussed in the Introduction.

The analysis of stochastic problems in random media requires sophisticated statistical processing and intensive computational simulations. The implementation strategies discussed for conductivity problems in Part I and the Introduction are equally applicable here. It could be repeated again and again that modern computational packages remain insufficient for conducting a comprehensive statistical analysis of composites, primarily due to the vast data requirements inherent in such studies incurring high computational costs, especially when the total computation time is accounted for. In contrast, the method of structural sums offers a computationally efficient alternative and can be successfully applied to the sizable datasets encountered in this paper.

### 6.2. Simulations of Dispersed Random Composites

In this section, we consider three algorithms designed to generate random configurations in the unitary torus $T^{2}={\left[0,1\right)}^{2}$ equipped with metric (28). These algorithms are coined as *R*, *T*, and *P*, respectively. Each of them produces a configuration of *N* disks with a fixed radius *r* distributed on the plane without overlaps, but they differ in the ways in which candidate centers are sampled and accepted. In what follows, we give a brief description of these algorithms.

#### 6.2.1. Variant R

The algorithm *R* corresponds to the classical procedure of RSA (*random sequential adsorption*) [64,65]. Random configurations are generated according to the following scheme. First, a point $a_{1}\in T^{2}$ is chosen uniformly at random, which defines the center of the first disk $K(a_{1},r)$. Next, a point $a_{2}\in T^{2}$ is selected. If $∥a_{1}-a_{2}∥<2r$, the point $a_{2}$ is rejected. The point is resampled until the following inequality

$$∥a_{1}-a_{2}∥\geq 2r$$

is met. In the next step, a point $a_{3}\in T^{2}$ is chosen. It is accepted only if

$$∥a_{3}-a_{j}∥\geq 2r,\;j=1,2.$$

Otherwise, $a_{3}$ is rejected and resampled. In the same way, for each subsequent point $a_{k}$, k=4,…,N, we require that the new center satisfies the non-overlapping condition with all previously accepted disks:

(54) $$∥a_{k}-a_{j}∥\geq 2r\;\mathrm{for}\;j=1,2,\ldots ,k-1.$$

As a result, we obtain a collection of *N* disks ${\left\{D\left(a_{k},r\right)\right\}}_{k=1}^{N}$ placed on the torus $T^{2}$ without overlaps. Detailed description of mechanisms for generating specific random disk configurations can be found in [64,65].

#### 6.2.2. Variant T

The goal is to generate four distinct datasets with target concentration values. The values of 0.1, 0.2, 0.3, and 0.4 are chosen. The concentration is defined by Equation (24). Introduce $d_{\min}=\min_{a_{k}\neq a_{m}}∥a_{k}-a_{m}∥$. For example, the minimum distance threshold $d_{\min}=0.01215$ for f=0.4. The latter threshold was selected according to a mathematical transformation of Formula (24) to achieve the desired concentration value f=0.4. Similarly, for concentration f=0.3, we have $d_{\min}=0.01052$; for concentration f=0.2, we have $d_{\min}=0.008593$; and for concentration f=0.1, we have $d_{\min}=0.006076$.

Then, we need to “clean” the data with an explicit goal to avoid overlapping. To this end, a cleaning procedure is activated when the distances between the interior points fall below this example threshold $d_{\min}<0.01215$. In such a case, an iterative process is activated that merges proximate points by taking their average until all pairs satisfy the distance criterion. For example, at f=0.4, the procedure amounts to following operations:

- Calculate pairwise Euclidean distances between all points;
- Locate the closest pair of points using the implemented algorithm;
- Activate cleaning when $d_{\min}<0.01215$;
- Replace the proximate points with their geometric center;
- The procedure is repeated until all pairs of points satisfy the distance criterion $d_{\min}\geq 0.01215$.

This iterative data cleaning process ensures a random distribution while preserving periodicity on a torus. The comprehensive statistical test for a sample concentration value of f=0.4 consists of 100 trials. According to the test results, the cleaning and generation procedures are consistent with their goals. Based on this, we make the following assumptions for the data cleaning procedure. Firstly, we assume that the final minimum distances should not exceed the threshold of 0.01215. Secondly, the achieved average concentration of 0.405 must demonstrate perfect agreement with the target value of f=0.4. The test results are presented in Table 1.

#### 6.2.3. Variant P

We proceed to discuss the procedure coined as *settling method* (or *deposition method*), because it mimics the physical process of settling overlapping grains or particles until mutual overlaps are removed. The settling method provides a constructive alternative to the classical RSA scheme. In contrast to RSA, where trials with overlaps are discarded, the settling method resolves overlaps through iterative spreading of all disks, allowing us to reach efficiently higher concentrations while keeping randomness in the spatial distribution of disk centers.

First, some configuration of *N* disks is generated with the disks allowed to overlap. If two disks $D_{i}$ and $D_{j}$ overlap, instead of deleting one of them, the corrective displacement δ to $a_{i},a_{j}$ is introduced by the rule $a_{i}\mapsto a_{i}-\frac{1}{2}\;\delta$, $a_{j}\mapsto a_{j}+\frac{1}{2}\;\delta$. After each correction on all overlapping disks, their coordinates are wrapped back onto the torus by the modulus of periods.

This iterative spreading procedure is repeated until the configuration of disks without overlaps is reached. Alternatively, the procedure could be repeated until a preset maximum number of iterations $t_{\max}$ is reached. The method converges to a final random configuration with all disks mutually disjoint. The final state corresponds to a random packing at the prescribed density without overlaps.

### 6.3. Results of Simulations

The simulation results are presented below, assuming that μ=1. The designation $\mu_{e}\equiv \mu_{J}\left(\varrho ,f\right)$ is used for the normalized effective shear modulus, where the subscript *J* corresponds to one of the protocols, i.e., J=P,R,T. According to (31), in the general form, $\mu_{J}$ has the form

(55) $$\mu_{J}\left(\varrho ,f\right)=\frac{1+A_{J}\left(\varrho ,f\right)}{1-\kappa A_{J}\left(\varrho ,f\right)}+O\left(f^{5}\right).$$

The explicit formulas for the coefficients $A_{J}\left(\varrho ,f\right)$ are given below. The dependence on the contrast parameter is presented for several concentrations.

(56) $$\begin{array}{c} A_{R}\left(\varrho ,0.1\right)=\varrho_{3}f+0.0038\varrho_{3}^{3}f^{2}+\left(\frac{1.991\varrho_{1}\varrho_{3}}{1+\varrho_{1}}-\left(0.1125+\frac{1.991}{1+\varrho_{1}}\right)\varrho_{3}^{2}+9.0733\varrho_{3}^{3}\right)f^{3}\\ +\left(\left(5.9528\varrho_{2}\varrho_{3}+\frac{13.2631\varrho_{3}^{2}}{1+\varrho_{1}}\right)\varrho_{1}-\left(15.3455+\frac{13.2631}{1+\varrho_{1}}\right)\varrho_{3}^{3}-0.0906\varrho_{3}^{4}\right)f^{4}, \end{array}$$

(57) $$\begin{array}{c} A_{R}\left(\varrho ,0.2\right)=\varrho_{3}f-0.0145\varrho_{3}^{3}f^{2}+\left(\frac{0.7813\varrho_{1}\varrho_{3}}{1+\varrho_{1}}-\left(0.0028+\frac{0.7813}{1+\varrho_{1}}\right)\varrho_{3}^{2}+4.2281\varrho_{3}^{3}\right)f^{3}\\ +\left(\left(1.4331\varrho_{2}\varrho_{3}+\frac{3.656\varrho_{3}^{2}}{1+\varrho_{1}}\right)\varrho_{1}-\left(3.3609+\frac{3.656}{1+\varrho_{1}}\right)\varrho_{3}^{3}-0.0928\varrho_{3}^{4}\right)f^{4}, \end{array}$$

(58) $$\begin{array}{c} A_{R}\left(\varrho ,0.3\right)=\varrho_{3}f+0.0215\varrho_{3}^{3}f^{2}+\left(\frac{0.3938\varrho_{1}\varrho_{3}}{1+\varrho_{1}}-\left(0.0536+\frac{0.3938}{1+\varrho_{1}}\right)\varrho_{3}^{2}+2.6224\varrho_{3}^{3}\right)f^{3}\\ +\left(\left(0.5896\varrho_{2}\varrho_{3}+\frac{1.4498\varrho_{3}^{2}}{1+\varrho_{1}}\right)\varrho_{1}-\left(0.9578+\frac{1.4498}{1+\varrho_{1}}\right)\varrho_{3}^{3}+0.0679\varrho_{3}^{4}\right)f^{4}, \end{array}$$

(59) $$\begin{array}{c} A_{R}\left(\varrho ,0.4\right)=\varrho_{3}f+0.0138\varrho_{3}^{3}f^{2}+\left(\frac{0.2036\varrho_{1}\varrho_{3}}{1+\varrho_{1}}-\left(0.0233+\frac{0.2036}{1+\varrho_{1}}\right)\varrho_{3}^{2}+1.7569\varrho_{3}^{3}\right)f^{3}\\ +\left(\left(0.3043\varrho_{2}\varrho_{3}+\frac{0.7001\varrho_{3}^{2}}{1+\varrho_{1}}\right)\varrho_{1}-\left(-0.0135+\frac{0.7001}{1+\varrho_{1}}\right)\varrho_{3}^{3}+0.0474\varrho_{3}^{4}\right)f^{4}, \end{array}$$

(60) $$\begin{array}{c} A_{T}\left(\varrho ,0.1\right)=\varrho_{3}f-0.0224\varrho_{3}^{3}f^{2}+\left(\frac{4.1724\varrho_{1}\varrho_{3}}{1+\varrho_{1}}-\left(-0.0750+\frac{4.1724}{1+\varrho_{1}}\right)\varrho_{3}^{2}+17.084\varrho_{3}^{3}\right)f^{3}\\ +\left(\left(16.046\varrho_{2}\varrho_{3}+\frac{36.505\varrho_{3}^{2}}{1+\varrho_{1}}\right)\varrho_{1}-\left(42.1002+\frac{36.505}{1+\varrho_{1}}\right)\varrho_{3}^{3}-1.1448\varrho_{3}^{4}\right)f^{4}, \end{array}$$

(61) $$\begin{array}{c} A_{T}\left(\varrho ,0.2\right)=\varrho_{3}f-0.013\varrho_{3}^{3}f^{2}+\left(\frac{1.6199\varrho_{1}\varrho_{3}}{1+\varrho_{1}}-\left(-0.0539+\frac{1.6199}{1+\varrho_{1}}\right)\varrho_{3}^{2}+7.1301\varrho_{3}^{3}\right)f^{3}\\ +\left(\left(3.2517\varrho_{2}\varrho_{3}+\frac{8.5578\varrho_{3}^{2}}{1+\varrho_{1}}\right)\varrho_{1}-\left(6.9732+\frac{8.5578}{1+\varrho_{1}}\right)\varrho_{3}^{3}-0.3749\varrho_{3}^{4}\right)f^{4}, \end{array}$$

(62) $$\begin{array}{c} A_{T}\left(\varrho ,0.3\right)=\varrho_{3}f-0.0189\varrho_{3}^{3}f^{2}+\left(\frac{0.9206\varrho_{1}\varrho_{3}}{1+\varrho_{1}}-\left(-0.0137+\frac{0.9206}{1+\varrho_{1}}\right)\varrho_{3}^{2}+4.5480\varrho_{3}^{3}\right)f^{3}\\ +\left(\left(1.4205\varrho_{2}\varrho_{3}+\frac{4.2749\varrho_{3}^{2}}{1+\varrho_{1}}\right)\varrho_{1}-\left(2.4282+\frac{4.2749}{1+\varrho_{1}}\right)\varrho_{3}^{3}-0.5347\varrho_{3}^{4}\right)f^{4}, \end{array}$$

(63) $$\begin{array}{c} A_{T}\left(\varrho ,0.4\right)=\varrho_{3}f-0.0503\varrho_{3}^{3}f^{2}+\left(\frac{0.5733\varrho_{1}\varrho_{3}}{1+\varrho_{1}}-\left(-0.0774+\frac{0.5733}{1+\varrho_{1}}\right)\varrho_{3}^{2}+3.0974\varrho_{3}^{3}\right)f^{3}\\ +\left(\left(0.7589\varrho_{2}\varrho_{3}+\frac{2.2534\varrho_{3}^{2}}{1+\varrho_{1}}\right)\varrho_{1}-\left(0.723+\frac{2.2534}{1+\varrho_{1}}\right)\varrho_{3}^{3}-0.374\varrho_{3}^{4}\right)f^{4}, \end{array}$$

(64) $$\begin{array}{c} A_{P}\left(\varrho ,0.1\right)=\varrho_{3}f+0.0511\varrho_{3}^{3}f^{2}+\left(\frac{4.3234\varrho_{1}\varrho_{3}}{1+\varrho_{1}}-\left(0.5600+\frac{4.3234}{1+\varrho_{1}}\right)\varrho_{3}^{2}+17.4621\varrho_{3}^{3}\right)f^{3}\\ +\left(\left(16.5948\varrho_{2}\varrho_{3}+\frac{37.5302\varrho_{3}^{2}}{1+\varrho_{1}}\right)\varrho_{1}-\left(41.7861+\frac{37.5302}{1+\varrho_{1}}\right)\varrho_{3}^{3}+2.255\varrho_{3}^{4}\right)f^{4}, \end{array}$$

(65) $$\begin{array}{c} A_{P}\left(\varrho ,0.2\right)=\varrho_{3}f+0.0018\varrho_{3}^{3}f^{2}+\left(\frac{1.7605\varrho_{1}\varrho_{3}}{1+\varrho_{1}}-\left(0.0759+\frac{1.7605}{1+\varrho_{1}}\right)\varrho_{3}^{2}+7.7548\varrho_{3}^{3}\right)f^{3}\\ +\left(\left(3.6094\varrho_{2}\varrho_{3}+\frac{9.6556\varrho_{3}^{2}}{1+\varrho_{1}}\right)\varrho_{1}-\left(7.4011+\frac{9.6556}{1+\varrho_{1}}\right)\varrho_{3}^{3}-0.3878\varrho_{3}^{4}\right)f^{4}, \end{array}$$

(66) $$\begin{array}{c} A_{P}\left(\varrho ,0.3\right)=\varrho_{3}f-0.0048\varrho_{3}^{3}f^{2}+\left(\frac{0.9661\varrho_{1}\varrho_{3}}{1+\varrho_{1}}-\left(0.0071+\frac{0.9661}{1+\varrho_{1}}\right)\varrho_{3}^{2}+4.5512\varrho_{3}^{3}\right)f^{3}\\ +\left(\left(1.3832\varrho_{2}\varrho_{3}+\frac{3.9887\varrho_{3}^{2}}{1+\varrho_{1}}\right)\varrho_{1}-\left(2.1935+\frac{3.9887}{1+\varrho_{1}}\right)\varrho_{3}^{3}+0.0140\varrho_{3}^{4}\right)f^{4}, \end{array}$$

(67) $$\begin{array}{c} A_{P}\left(\varrho ,0.4\right)=\varrho_{3}f-0.0092\varrho_{3}^{3}f^{2}+\left(\frac{0.5474\varrho_{1}\varrho_{3}}{1+\varrho_{1}}-\left(0.0107+\frac{0.5474}{1+\varrho_{1}}\right)\varrho_{3}^{2}+2.9300\varrho_{3}^{3}\right)f^{3}\\ +\left(\left(0.6345\varrho_{2}\varrho_{3}+\frac{1.9763\varrho_{3}^{2}}{1+\varrho_{1}}\right)\varrho_{1}-\left(0.1715+\frac{1.9763}{1+\varrho_{1}}\right)\varrho_{3}^{3}-0.1053\varrho_{3}^{4}\right)f^{4}, \end{array}$$

The following remark clarifies the dependence of $\mu_{J}\left(\varrho ,f\right)$ on concentration. Each term $A_{J}\left(\varrho ,f\right)$ was decomposed into a series of powers in *f*. The latter was reduced to a fourth-order polynomial with the coefficients explicitly written through the contrast parameters given in symbolic form and the numerically computed structural sums. The structural sums were simulated 100 times according to each algorithm P,R,T. Finally, their mean was taken. The numerical values in the above formulas contain these mean values. Every algorithm depends on the concentration *f* due to the condition of no overlaps.

Since the numerical coefficients of $A_{J}\left(\varrho ,f\right)$ are given as the powers of *f*, every set of structural sums is also dependent on *f*. This dependence may change with modifications of the algorithm. For example, the introduction of security disks [29] artificially increases the parameter *f* in $A_{J}\left(\varrho ,f\right)$. Hence, the numerical values in (56) and (67) tacitly depend on the concentration given numerically f=0.1,0.2,0.3,0.4. At the same time, $A_{J}\left(\varrho ,f\right)$ depends analytically on the powers of *f*.

There is a discrepancy between the theoretical and simulated values of the coefficients. Consider the term $A^{\left(2\right)}$ given by (34) symbolically for any set of centers (2). The theoretical equation $A^{\left(2\right)}=0$ is satisfied if the composite is macroscopically isotropic. The algorithms P,R,T are theoretically isotropic. However, even after 100 simulations of the N=100 disks, their mean may not be perfectly isotropic. The coefficients 0.0038, −0.0145, 0.0215, *…* in $\varrho_{3}f$ or their squares can be considered as the degree of deviation from perfect isotropy. In the next section a simple criterion for selecting the best approximation that gives a minimal deviation from the perfect theoretical values of the coefficients is formulated.

An advanced statistical analysis was performed in [1,66] for antiplane shear. An analogous analysis will be made in a separate paper together with machine learning investigations.

## 7. Critical Index for the Effective Shear Modulus of Composite with Hard Inclusions

The normalized effective shear modulus $\mu_{e}\left(f\right)$ of a composite made with hard inclusions could be found in a convenient polynomial form, with the material parameters set to the following values:

(68) $$\kappa =\varrho_{1}=\varrho_{2}=\varrho_{3}=1.$$

The protocols used for the construction of approximation polynomials in small and moderate *f* are compatible with the percolation regime of touching disks for large *f*. Three different sets containing 100 disks were considered for different concentrations 0.1, 0.2, 0.3, and 0.4. The initial state was generated by RSA. Three different protocols *R*, *T*, *P* found the final state. We substitute (68) into (55) and (56) and (67). Then, up to $O(f^{5})$,

(69) $$\mu_{R}\left(1,0.1\right)=\frac{1+f+0.0038f^{2}+8.9608f^{3}-9.4832f^{4}}{1-f-0.0038f^{2}-8.9608f^{3}+9.4832f^{4}},$$

(70) $$\mu_{R}\left(1,0.2\right)=\frac{1+f-0.0145f^{2}+4.2253f^{3}-0.5876f^{4}}{1-f+0.0145f^{2}-4.2253f^{3}+0.5876f^{4}},$$

(71) $$\mu_{R}\left(1,0.3\right)=\frac{1+f+0.0215f^{2}+2.5687f^{3}+0.2891f^{4}}{1-f-0.0215f^{2}-2.5687f^{3}-0.2891f^{4}},$$

(72) $$\mu_{R}\left(1,0.4\right)=\frac{1+f+0.0138f^{2}+1.7336f^{3}+0.6695f^{4}}{1-f-0.0138f^{2}-1.7336f^{3}-0.6695f^{4}},$$

(73) $$\mu_{T}\left(1,0.1\right)=\frac{1+f-0.0224f^{2}+17.1591f^{3}-24.9094f^{4}}{1-f+0.0224f^{2}-17.1591f^{3}+24.9094f^{4}}$$

(74) $$\mu_{T}\left(1,0.2\right)=\frac{1+f-0.0130f^{2}+7.1840f^{3}-0.8448f^{4}}{1-f+0.0130f^{2}-7.1840f^{3}+0.8448f^{4}},$$

(75) $$\mu_{T}\left(1,0.3\right)=\frac{1+f-0.0189f^{2}+4.5616f^{3}-0.1218f^{4}}{1-f+0.0189f^{2}-4.5616f^{3}+0.1218f^{4}},$$

(76) $$\mu_{T}\left(1,0.4\right)=\frac{1+f-0.0503f^{2}+3.1748f^{3}+0.4207f^{4}}{1-f+0.0503f^{2}-3.1748f^{3}-0.4207f^{4}},$$

(77) $$\mu_{P}\left(1,0.1\right)=\frac{1+f+0.0511f^{2}+16.902f^{3}-22.9362f^{4}}{1-f-0.0511f^{2}-16.902f^{3}+22.9362f^{4}}$$

(78) $$\mu_{P}\left(1,0.2\right)=\frac{1+f+0.0018f^{2}+7.6789f^{3}-0.5701f^{4}}{1-f-0.0018f^{2}-7.6789f^{3}+0.5701f^{4}},$$

(79) $$\mu_{P}\left(1,0.3\right)=\frac{1+f-0.0048f^{2}+4.5440f^{3}+0.5868f^{4}}{1-f+0.0048f^{2}-4.5440f^{3}-0.5868f^{4}},$$

(80) $$\mu_{P}\left(1,0.4\right)=\frac{1+f-0.0092f^{2}+2.9193f^{3}+0.9921f^{4}}{1-f+0.0092f^{2}-2.9193f^{3}-0.9921f^{4}}.\;$$

In the following, the shortened designation for the effective shear modulus $\mu_{e}\equiv \mu_{J}\left(1,f\right)$ is used. The effective shear modulus is divergent as $f\to f_{c}$, where $f_{c}$ stands for the continuum percolation threshold, so that

$$\mu_{e}≃B{\left(f_{c}-f\right)}^{-s},$$

where *B* stands for the critical amplitude and $f_{c}=\frac{\pi }{\sqrt{12}}\approx 0.9069$.

The coefficient *B* and its approximations below, as well as the shear modulus $\mu_{e}$ and its approximations, are normalized to the unit shear modulus of the host, μ=1. Therefore, they are non-dimensional quantities.

Our goal is to determine the critical index *s* for the effective shear modulus from the coefficients of the approximate polynomials and $f_{c}$. The expected value for the critical index s≈1.3 [29,33].

Approximations (69)–(80) for the normalized effective shear modulus are found using simulations for small and moderate *f*. These expressions can be expanded into Taylor series and approximated by polynomials

(81) $$\mu_{e,k}=\sum_{n=0}^{k}c^{\left(n\right)}f^{n},$$

where the coefficients $c^{\left(n\right)}$ are expressed through the coefficients of (69)–(80). We have $c^{\left(0\right)}=1$, $c^{\left(1\right)}=2$, $c^{\left(2\right)}=2\left(1+A^{\left(2\right)}\right)$, etc. The coefficients $A^{\left(2\right)}$ are the coefficients of the numerators of (69)–(80) in $f^{2}$.

We employ the Borel-type transformation of the following form:

(82) $$\psi_{k,b}\left(f\right)=\sum_{n=0}^{k}\frac{c^{\left(n\right)}}{{\left(\Gamma (1+n)\right)}^{b}}\;f^{n}\;,$$

and apply it to the original truncated power series (81) with k=4 to improve its convergence [67,68]. The parameter *b* represents the discrete number of iterations to be applied to a Borel transformation. It was considered equal to 0 or 1 in the current work. When b=0, we return to the original polynomial approximations. When b=1, we arrive at the celebrated Borel transformation.

The critical amplitude *B* after the inverse Borel-type transformation is approximated as follows:

(83) $$B_{k,b}\left(s\right)=f_{c}^{s}\;C_{k,b}\left(s\right){\left(\Gamma \left(1+s\right)\right)}^{b}\;,$$

where

(84) $$C_{k,b}\left(s\right)={\left({\left({\left(A_{1,b}{\left(s\right)}^{2}+A_{2,b}\left(s\right)\right)}^{3/2}+A_{3,b}\left(s\right)\right)}^{4/3}+\ldots +A_{k,b}\left(s\right)\right)}^{s_{k,b}/k}\;,$$

is expressed [68] from the approximant

(85) $$\psi_{k,b}^{*}\left(f\right)={\left({\left({\left({\left(1+A_{1,b}z\left(f\right)\right)}^{2}+A_{2,b}z{\left(f\right)}^{2}\right)}^{3/2}+A_{3,b}z{\left(f\right)}^{3}\right)}^{4/3}+\ldots +A_{k,b}z{\left(f\right)}^{k}\right)}^{s_{4,b}/k}\;,$$

for the iterated roots, with $z\left(f\right)=\frac{f}{f_{c}-f}.$ In the limit $f\to f_{c}$,

(86) $$\psi_{k,b}^{*}\left(f\right)≃f_{c}^{s_{k,b}}{\left(f_{c}-f\right)}^{-s_{k,b}}\;C_{k,b}\left(s_{k,b}\right).$$

The divergence in (86) is like a power law, and $s_{k,b}\approx s$ gives the approximate values of the index *s* for each *k* and *b*. “Amplitudes” $A_{k,b}$ are determined by asymptotic matching of formulas (85) and (82) at f→0.

To find the approximations for the index, a minimal difference condition is imposed on the critical amplitudes, see [69] and the works cited therein,

(87) $$B_{k,0}\left(s\right)-B_{k-1,0}\left(s\right)=0,\;s=s_{k,0}=s_{k-1,0},$$

with k=4. It does not require the Borel transformation at all. The critical index for the prime quantity of interest, the effective shear modulus, is found by solving Equation (87). It is also an optimization parameter that ensures the convergence of the resummation procedure. Even without introducing any new control parameters, it appears to be a way to control the convergence of the sequence of critical amplitudes through the critical index by itself.

Otherwise, one can apply the hybrid minimal sensitivity difference condition

(88) $$\frac{\partial B_{k,1}\left(s\right)}{\partial s}-\frac{\partial B_{k,0}\left(s\right)}{\partial s}=0,\;s=s_{k,1}=s_{k,0},$$

which minimizes the difference of the two sensitivities with k=4. Equation (88) formalizes the requirement of sensitivity independence of the number of iterations. Solving Equations (87) and (88), we find estimates for the critical indices. Equations (87) and (88) in each case reported in the tables give unique solutions. Thus, the critical index *s* is deduced from the coefficients $c^{\left(n\right)}$ of the approximate rational approximations reduced to expansion (81) and from the threshold $f_{c}$.

The following rational approximations to the shear modulus were computed as explained above. The polynomial corresponding to the *R* protocol up to $O(f^{5})$ can be obtained from (69)–(72)

$$\mu_{e,4}^{R}\left(1,f\right)=1+2f+2.01227f^{2}+10.7687f^{3}+13.806f^{4}.$$

Equations (73)–(76) give

$$\mu_{e,4}^{P}\left(1,f\right)=1+2f+2.0195f^{2}+18.0611f^{3}+20.3269f^{4}.$$

Equations (77)–(80) produce the approximating polynomial corresponding to the *P* protocol

$$\mu_{e,4}^{T}\left(1,f\right)=1+2f+1.94768f^{2}+17.9351f^{3}+18.4812f^{4}.$$

We also consider the average taken over all *R*, *P*, and *T* protocols for all concentrations, with the number of components M=12:

$$\mu_{e,4}^{\left\{12\right\}}\left(f\right)=1+2f+1.99315f^{2}+15.5883f^{3}+17.5381f^{4}.$$

Average for all protocols without taking into account polynomials computed at concentration 0.4, with the number of components M=9:

$$\mu_{e,4}^{\left\{9\right\}}\left(f\right)=1+2f+2.00103f^{2}+18.3986f^{3}+19.1828f^{4}.$$

Average for all protocols without taking into account computations at concentrations 0.3 and 0.4, with the number of components M=6:

$$\mu_{e,4}^{\left\{6\right\}}\left(f\right)=1+2f+2.00228f^{2}+22.7079f^{3}+20.8729f^{4}.$$

Average for all protocols for concentration 0.1, with the number of components M=3:

$$\mu_{e,4}^{\left\{3\right\}}\left(f\right)=1+2f+2.02171f^{2}+30.7247f^{3}+21.2105f^{4}.$$

The following results can be found with relative ease for the critical index *s* for the *R*, *P*, and *T* protocols considered separately. The results are shown in Table 2.

The results found from the *T* protocol seem to be the most consistent among all protocols when it comes to index calculations, as it provides the tightest bounds for the index. However, for small concentrations it is inferior to the other protocols. It seems desirable to have an approximation valid for small and high concentrations as well.

It is possible to formulate a simple criterion for selecting the best approximating polynomial. Let us select the polynomial that gives a minimal deviation from the theoretical value of the coefficient $c^{\left(2\right)}=2$.

The latter simple criterion prefers the average for all protocols computed without the polynomials computed at f=0.4, for the polynomial found at M=9, as shown in Table 3.

In the preferred case just discussed above, the optimal conditions give *s* in the range from $s_{min}=1.27434$ to $s_{max}=1.40231$, as shown in Table 4.

**Figure 4:**
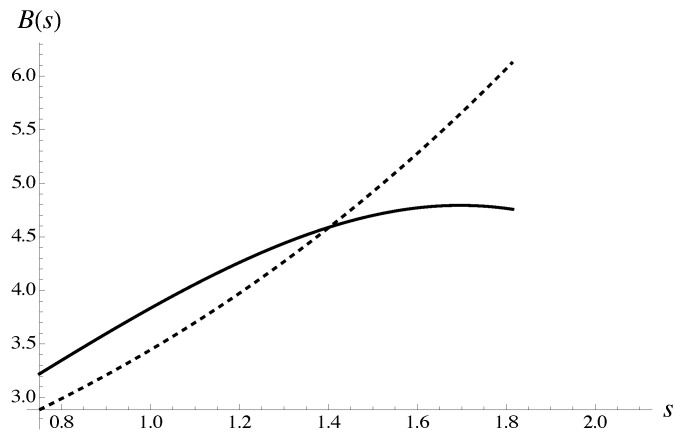
The solution to Equation (87) is explained graphically. Expression $B_{4,0}\left(s\right)$ is shown with a solid line. Expression $B_{3,0}\left(s\right)$ is shown with a dashed line. The approximations for amplitudes follow, generally speaking, from asymptotic equivalence of the corresponding iterated root approximants with average polynomial $\mu_{e,4}^{\left\{9\right\}}$. The latter is computed for all protocols without taking into account the polynomials at concentration 0.4.

The solution to Equation (87)

$$B_{4,0}\left(s\right)=B_{3,0}\left(s\right),\;s=s_{4,0}=s_{3,0},$$

is presented graphically in Figure 4. Explicit expressions $B_{3,0}\left(s\right)$ and $B_{4,0}\left(s\right)$ follow from the corresponding root approximants. The parameters for the approximants are derived from the average polynomial $\mu_{e,4}^{\left\{9\right\}}$ calculated for all protocols without polynomials at concentration 0.4.

**Figure 5:**
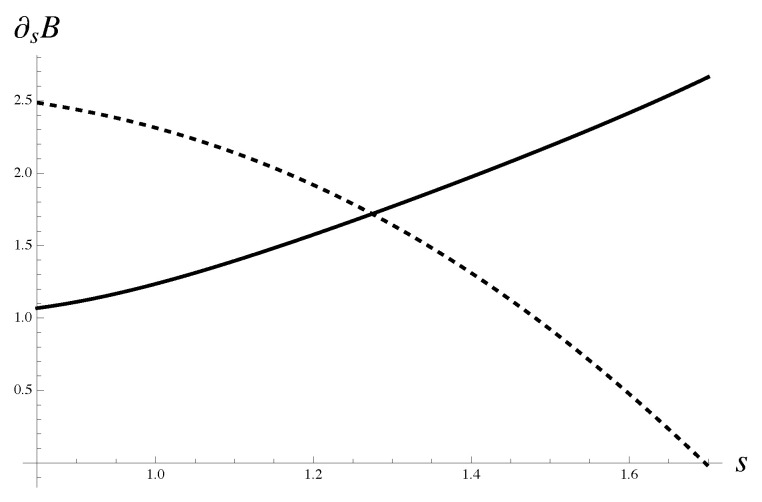
The solution to Equation (88) is illustrated graphically. Expression $\frac{\partial B_{4,1}\left(s\right)}{\partial s}$ is shown with solid line. Expression $\frac{\partial B_{4,0}\left(s\right)}{\partial s}$ is shown with dashed line. The approximations for amplitudes dependent on index *s* follow from asymptotic equivalence of the corresponding iterated root approximants with average polynomial $\mu_{e,4}^{\left\{9\right\}}$. The latter is computed for all protocols without taking into account the polynomials at concentration 0.4.

The solution to Equation (88)

$$\frac{\partial B_{4,1}\left(s\right)}{\partial s}=\frac{\partial B_{4,0}\left(s\right)}{\partial s},\;s=s_{4,1}=s_{4,0},$$

is presented graphically in Figure 5. Explicit expressions $\frac{\partial B_{4,0}\left(s\right)}{\partial s}$, $\frac{\partial B_{4,1}\left(s\right)}{\partial s}$ follow from the corresponding root approximants. The approximant parameters are derived from the average polynomial $\mu_{e,4}^{\left\{9\right\}}$ calculated for all protocols without polynomials at concentration 0.4, M=9.

From the expression (85), with b=0 and with fixed critical index

$$s=\frac{s_{min}+s_{max}}{2}=1.33833,$$

the effective shear modulus for all *f* can be approximated by the iterated root

(89) $$\mu_{e}^{*}={\left[\frac{6.6104f^{4}}{{\left(0.9069-f\right)}^{4}}+{\left(\frac{26.55f^{3}}{{\left(0.9069-f\right)}^{3}}+{\left({\left(\frac{1.3553f}{0.9069-f}+1\right)}^{2}-\frac{0.8725f^{2}}{{\left(0.9069-f\right)}^{2}}\right)}^{\frac{3}{2}}\right)}^{\frac{4}{3}}\right]}^{0.3346},$$

while k=4, b=0. From the latter formula, we estimate the critical amplitude B≈3.9486.

**Figure 6:**
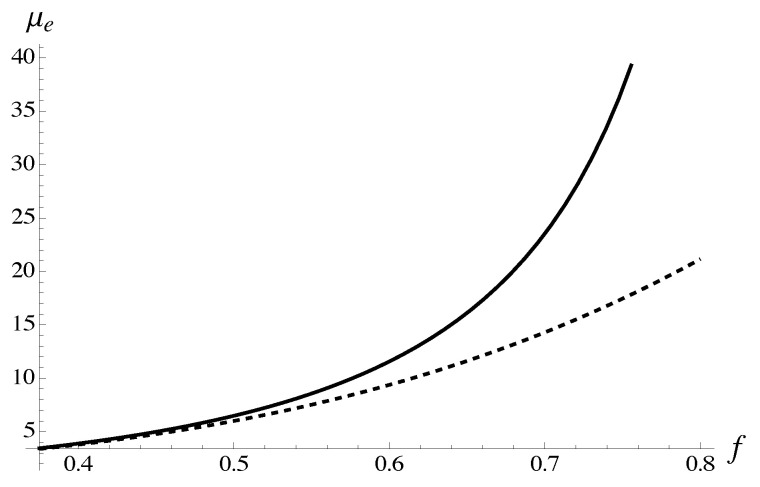
Formula (89) (solid), is compared with the average polynomial $\mu_{e,4}^{\left\{9\right\}}$ computed for all protocols without taking into account polynomials at concentration 0.4. The latter is shown with dashed line.

The expression (89) can be compared with the average for all protocols calculated without polynomials at concentration 0.4, as shown in Figure 6. The resummed expression already deviates from the averaged polynomial $\mu_{e,4}^{\left\{9\right\}}$ as f≈0.4, as the transition to the critical regime sets in.

Most of the results presented in Table 4 for the critical index *s* are reasonably good. Even without resorting to the criterion suggested above, one can safely estimate s≈1.292 based on the last column of Table 4. The average of three different protocols applied at small concentrations gives the most consistent extrapolation to the critical region, even without bringing the best estimate of the coefficient $c^{\left(2\right)}$ in (81).

## 8. Conclusions

The key message of this paper is the urgency to respect the principles of homogenization theory when determining the effective elastic properties of composite materials. Hill’s self-consistent conception [14,63] was developed on intuitive physical grounds prior to the establishment of formal homogenization theory. It has often been applied for arbitrary domains *V* without rigorous justification. In this work, we established a theoretical foundation for Hill’s approach when *V* coincides with a fundamental domain. In contrast, when this condition is not met, self-consistent approximations yield effective constants only up to $O(f^{2})$, valid only for some dilute clusters [13,17].

This insight totally condemns some methodologies as misleading.

Hill’s intuitive physical conception was revised with the application of *a*RVE theory to a random 2D elastic composite, focusing on macroscopically isotropic composites. Realization of the proposed strategy meant computing the shear and bulk moduli using structural sums. The resulting power series expansion in terms of the inclusion concentration *f* contained the coefficients exactly written through the location of inclusions and their elastic constants up to $O(f^{5})$. The coefficients were computed using three Monte-Carlo protocols.

Theoretically, we considered only a single geometric probabilistic distribution, corresponding to the uniform distribution of non-overlapping disks, but implemented it through different protocols *R*, *T*, and *P* for varying concentrations. The numerical simulations led to various analytical formulas, which are presented in Section 6 and Section 7. The resummation procedure was employed to transform polynomial expressions into rational functions with fractional powers or iterated roots. The transformation produced reasonable estimates for the critical index for the shear modulus, as well as compact formulas for the effective constants valid for all *f*. The latter insights justify all our theoretical and computational efforts. For a given experimental sample, the entire scheme could be employed along the lines of [61,70,71]. The following topics will be discussed in future extensions of this research. An advanced statistical analysis was performed in [1,66] for antiplane shear. A similar analysis will be performed for the problem at hand. We intend to complement it with some machine learning techniques. The computation of the effective bulk modulus is deferred to a forthcoming Part III.

## Figures and Tables

**Table 1:** Statistical summary of tests performed on synthetic cleaning data across 100 trials for target concentration f=0.4.

Parameter	Minimum	Maximum	Mean	Std Dev
Initial point count	273	450	336.5	32.8
Final point count	75	115	92.7	8.9
Point reduction	72.5%	74.4%	72.4%	2.1%
Final minimum distance	0.01224	0.01402	0.01257	0.15
Achieved concentration (*f*)	0.316	0.511	0.405	0.038

**Table 2:** Critical index *s* (k=4).

*s*	*R* Protocol	*P* Protocol	*T* Protocol
Minimal Difference (87)	1.50628	1.43399	1.39844
Minimal Sensitivity Difference (88)	1.25551	1.29169	1.26844

**Table 3:** Expansion coefficient $c^{\left(2\right)}$. Exact $c^{\left(2\right)}=2$.

	Average, M=12	Average, M=9	Average, M=6	Average, M=3
$c^{\left(2\right)}$	1.99315	2.00103	2.00228	2.02171

**Table 4:** Critical index *s* (k=4).

*s*	Aver., M=12	Aver., M=9	Aver., M=6	Aver., M=3
Min. Diff. (87)	1.43417	1.40231	1.35931	1.28867
Min. Sens. Diff. (88)	1.26628	1.27434	1.28509	1.29518

## Data Availability

The original contributions presented in the study can be found in the article. Further inquiries can be directed to the corresponding author.

## Appendix A

Let $\omega_{j}$ (j=1,2) be the fundamental translation vectors expressed by complex numbers that generate the lattice $Q=\{P\equiv m_{1}\omega_{1}+m_{2}\omega_{2},\;m_{1,2}\in Z\}$, where Z={0,±1,±2,…}. It is assumed that $\omega_{1}>0$, Im ω2>0, and the area of the fundamental domain is normalized to unity, so that ω1Im ω2= 1. Filshtinsky in Appendix 2, [25] introduced a new meromorphic function ${℘}_{1}\left(z\right)$ similar to the Weierstrass elliptic functions ℘(z) and ζ(z). Its derivative

(A1) $${℘}_{1}^{\prime }\left(z\right)=-2\sum_{\left(m_{1},m_{2}\right)\in Z^{2}\setminus \left(0,0\right)}\left[\frac{\bar{P}}{{\left(z-P\right)}^{3}}+\frac{\bar{P}}{P^{3}}\right],$$

is introduced in the form of double series. The series (A1) converges absolutely and almost uniformly for z∈C∖Q. The function (A1) for the hexagonal array was first introduced by Natanzon [7].

The Natanzon–Filshtinsky function (A1) is closely related to the Eisenstein–Natanzon–Filshtinsky function introduced in [33] (see formula A.3 below),

(A2) $$E_{3}^{\left(1\right)}\left(z\right)=\sum_{m_{1},m_{2}\in Z}\frac{\bar{z-P}}{{\left(z-P\right)}^{3}}=\bar{z}E_{3}\left(z\right)+\frac{1}{2}{℘}_{1}^{\prime }\left(z\right)+S_{3}^{\left(1\right)},$$

where the classical Eisenstein function [72], represented by the absolutely convergent series,

(A3) $$E_{3}\left(z\right)=\sum_{m_{1},m_{2}\in Z}\frac{1}{{\left(z-P\right)}^{3}}=-\frac{1}{2}{℘}^{\prime }\left(z\right),$$

is used. The constant $S_{3}^{\left(1\right)}$ is introduced by the conditionally convergent series, which remains undefined at this step,

(A4) $$S_{3}^{\left(1\right)}=\sum_{\left(m_{1},m_{2}\right)\in Z^{2}\setminus \left(0,0\right)}\frac{\bar{P}}{P^{3}}.$$

The function $E_{3}^{\left(1\right)}\left(z\right)$ for the hexagonal fundamental domain $Q_{0,0}$ was defined in [56] with $S_{3}^{\left(1\right)}=\frac{\pi }{2}$.

Filshtinsky derived a general formula for ${℘}_{1}\left(z\right)$ [25] (see formula (20) from Appendix 2). The formula for the derivative in the case of square arrays is written as follows:

(A5) $$\pi {℘}_{1}^{\prime }\left(z\right)=\frac{1}{2}{℘}^{\prime \prime }\left(z\right)+{\left[\zeta \left(z\right)℘\left(z\right)\right]}^{\prime }+C.$$

The constant *C* was written in the form $C=\frac{1}{12}g_{4}^{*}-\frac{5}{8}g_{4}$, in our designations $g_{4}=16S_{4}$ and $g_{4}^{*}=\frac{15}{4}g_{4}=60S_{4}$. Hence, $C=-5S_{4}$. Using the identities borrowed from the theory of elliptic functions, ${℘}^{\prime \prime }\left(z\right)=6{℘}^{2}\left(z\right)-30S_{4}$ and $\zeta^{\prime }\left(z\right)=-℘\left(z\right)$, we write (A5) in the form

(A6) $${℘}_{1}^{\prime }\left(z\right)=\frac{1}{\pi }\left[\frac{1}{3}{℘}^{\prime \prime }\left(z\right)+\zeta \left(z\right){℘}^{\prime }\left(z\right)-10S_{4}\right].$$

Substitution of (A6) into (A2) yields

(A7) $$E_{3}^{\left(1\right)}\left(z\right)=\bar{z}E_{3}\left(z\right)+\frac{1}{\pi }E_{4}\left(z\right)-\frac{1}{\pi }\zeta \left(z\right)E_{3}\left(z\right)-\frac{5}{\pi }S_{4}+S_{3}^{\left(1\right)},$$

where $E_{4}\left(z\right)=\frac{1}{6}{℘}^{\prime \prime }\left(z\right)$ [12] and $S_{4}=\frac{\Gamma^{8}\left(\frac{1}{4}\right)}{2^{6}\cdot 3\cdot 5\pi^{2}}\approx 3.15121$ [33].

We now proceed to check if

(A8) $$F_{3}^{\left(1\right)}\left(z\right):=\frac{1}{\pi }E_{4}\left(z\right)-\frac{1}{\pi }\zeta \left(z\right)E_{3}\left(z\right)-\frac{5}{\pi }S_{4},$$

from (A7) is analytic at z=0.

Consider the lattice sums

(A9) $$S_{2j}=\sum_{\left(m_{1},m_{2}\right)\in Z^{2}\setminus \left(0,0\right)}\frac{1}{P^{2j}},\;j=1,2,3,\ldots .$$

The sum $S_{2}$ converges only conditionally. Hence, it must be defined, and $S_{2j}=0$ (j∈Z+1) is set for the square array. $S_{2}=0$ must be assumed for the computations of effective constants [56]. From the condition of macroscopic isotropy one can derive that $B_{2}=0$ in (46). Using the expansions [12,73]

(A10) $$\zeta \left(z\right)=\frac{1}{z}-S_{4}z^{3}-\sum_{j=2}^{\infty }S_{4j}z^{4j-1},$$

(A11) $$E_{3}\left(z\right)=-\frac{1}{2}{℘}^{\prime }\left(z\right)=\frac{1}{z^{3}}-3S_{4}z-\frac{1}{2}\sum_{j=2}^{\infty }\left(4j-1\right)\left(4j-2\right)S_{4j}z^{4j-3}$$

and

(A12) $$E_{4}\left(z\right)=\frac{1}{6}{℘}^{\prime \prime }\left(z\right)=\frac{1}{z^{4}}+S_{4}+\frac{1}{6}\sum_{j=2}^{\infty }\left(4j-1\right)\left(4j-2\right)\left(4j-3\right)S_{4j}z^{4j-4}$$

we get

(A13) $$F_{3}^{\left(1\right)}\left(z\right)=\frac{1}{\pi }\left[\frac{1}{z^{4}}+S_{4}-\left(\frac{1}{z}-S_{4}z^{3}\right)\left(\frac{1}{z^{3}}-3S_{4}z\right)\right]-\frac{5}{\pi }S_{4}+O\left(z^{4}\right)=O\left(z^{4}\right),\;\mathrm{as}\;z\to 0.$$

In particular,

(A14) $$\begin{array}{c} F_{3}^{\left(1\right)}\left(0\right)=0. \end{array}$$
